# ALCAT1-mediated abnormal cardiolipin remodelling promotes mitochondrial injury in podocytes in diabetic kidney disease

**DOI:** 10.1186/s12964-023-01399-4

**Published:** 2024-01-10

**Authors:** Yiqun Hao, Yanqin Fan, Jun Feng, Zijing Zhu, Zilv Luo, Hongtu Hu, Weiwei Li, Hongxia Yang, Guohua Ding

**Affiliations:** Division of Nephrology, Renmin Hospital of Wuhan University, 238 Jiefang Rd, Wuhan, Hubei 430060 China

**Keywords:** ALCAT1, Cardiolipin remodelling, Oxidized cardiolipin, Mitochondrial dysfunction, Podocyte injury, Diabetic kidney disease

## Abstract

**Background:**

Cardiolipin (CL) plays a critical role in maintaining mitochondrial membrane integrity and overall mitochondrial homeostasis. Recent studies have suggested that mitochondrial damage resulting from abnormal cardiolipin remodelling is associated with the pathogenesis of diabetic kidney disease (DKD). Acyl-coenzyme A:lyso-cardiolipin acyltransferase-1 (ALCAT1) was confirmed to be involved in the progression of Parkinson’s disease, diet-induced obesity and other ageing-related diseases by regulating pathological cardiolipin remodelling. Thus, the purpose of this investigation was to determine the role of ALCAT1-mediated CL remodelling in DKD and to explore the potential underlying mechanism.

**Methods:**

In vivo study, the mitochondrial structure was examined by transmission electron microscopy (TEM). The colocalization of ALCAT1 and synaptopodin was evaluated by double immunolabelling. Western blotting (WB) was performed to assess ALCAT1 expression in glomeruli. Lipidomics analysis was conducted to evaluate the composition of reconstructed cardiolipins. In vitro study, the lipidomics, TEM and WB analyses were similar to those in vivo. Mitochondrial function was evaluated by measuring the mitochondrial membrane potential (MMP) and the production of ATP and ROS.

**Results:**

Here, we showed that increased oxidized cardiolipin (ox-CL) and significant mitochondrial damage were accompanied by increased ALCAT1 expression in the glomeruli of patients with DKD. Similar results were found in db/db mouse kidneys and in cultured podocytes stimulated with high glucose (HG). ALCAT1 deficiency effectively prevented HG-induced ox-CL production and mitochondrial damage in podocytes. In contrast, ALCAT1 upregulation enhanced ox-CL levels and podocyte mitochondrial dysfunction. Moreover, treatment with the cardiolipin antioxidant SS-31 markedly inhibited mitochondrial dysfunction and cell injury, and SS-31 treatment partly reversed the damage mediated by ALCAT1 overexpression. We further found that ALCAT1 could mediate the key regulators of mitochondrial dynamics and mitophagy through the AMPK pathway.

**Conclusions:**

Collectively, our studies demonstrated that ALCAT1-mediated cardiolipin remodelling played a crucial role in DKD, which might provide new insights for DKD treatment.

Video Abstract

**Supplementary Information:**

The online version contains supplementary material available at 10.1186/s12964-023-01399-4.

## Introduction

Various microvascular complications are associated with diabetes, among which diabetic kidney disease is the most severe and prevalent. Diabetic kidney disease (DKD) is the leading cause of end-stage renal disease (ESRD) [1, 2]. Podocytes are considered a crucial component of the glomerular filtration barrier. Podocyte injury is highly correlated with the progression of DKD, as manifested by proteinuria [3]. While it is well established that podocyte injuries contribute to DKD, there are currently limited treatment options available [4–7]. Therefore, identifying new therapeutic targets to improve kidney function in patients with DKD is an urgent priority.

Mitochondria are critical cell organelles that generate energy and maintain cell homeostasis, particularly in energy-demanding tissues such as the kidney. Mitochondrial malfunction induced by hyperglycaemia leads to oxidative damage and cell apoptosis and is considered to contribute to the progression of DKD [8–12]. However, the mechanisms regulating mitochondrial abnormalities in DKD are not yet fully understood. As the signature phospholipid of mitochondria, cardiolipin (CL) is exclusively present in the mitochondrial membrane, and is essential for mitochondrial functions, including membrane structure, dynamics, and mitophagy [13–15].

Cardiolipin biosynthesis begins with glycerol-3-phosphate, which undergoes a series of reactions culminating in the formation of cytidinediphosphate-diacylglycerol and phosphatidylglycerol phosphate at the inner mitochondrial membrane and condensation to form cardiolipin. The condensed cardiolipin requires remodelling to form mature cardiolipin. There are two main pathways of cardiolipin remodelling. The first pathway involves transacylation, where acyl groups are transferred from phosphatidylcholine or phosphatidylethanolamine to CL. This process is partially catalyzed by tafazzin (TAZ), a transacylase enzyme. The alternative pathway consists of deacylation by phospholipase A2, which converts CL into lysocardiolipin. Subsequently, two acyl-CoA-dependent lysocardiolipin acyltransferases are responsible for reacylating lysocardiolipin back into CL. These two enzymes are acyl-CoA:lysocardiolipin acyltransferase (ALCAT1) and monolysocardiolipin acyltransferase (MLCLAT) respectively. Related research shows that in comparison to MLCLAT, ALCAT1 does not exhibit a preference for a substrate [15–18]. Thus, due to its low substrate specificity, ALCAT1 expression and function are aberrantly altered under stress conditions to abnormally reconstitute cardiolipin using very long-link unsaturated fatty acids as substrates. Abnormally remodelled CL has a high content of unsaturated fatty acids. This, coupled with the fact that cardiolipin is located in the mitochondrial membrane, where ros is produced, results in it being highly susceptible to oxidation by ROS, generating oxidised CL. The oxidised cardiolipin produced can in turn further contribute to its own peroxidation, thus triggering the process of self-destruction [19]. Oxidized CL has been confirmed to lead to mitochondrial dysfunction and cell apoptosis [20, 21]. Abnormalities in CL composition and content have been implicated in the pathogenesis of obesity, type 2 diabetes, and other ageing-related diseases [15, 22]. Ducasa et al. confirmed that inhibition of cardiolipin peroxidation with elamipretide (SS-31) improved DKD in db/db mice and prevented podocyte injury in vitro [23]. It is speculated that reducing CL peroxidation by inhibiting abnormal CL remodelling may alleviate the progression of DKD. Hence, identification of the key molecules regulating CL remodelling in podocytes may offer fresh perspectives for the treatment of DKD.

The effect of ALCAT1 on cardiolipin remodelling in a number of diseases has also been reported in the literature. Several ageing-related diseases, such as obesity, type 2 diabetes, and cardiovascular diseases, have been linked to ALCAT1 activity [16, 24, 25]. Recent studies reported that ALCAT1 expression was potently upregulated in myocardial infarction, linking myocardial hypoxia to oxidative stress and mitochondrial dysfunction [19]. Knockdown of the ALCAT1 gene or pharmacological inhibition of the ALCAT1 enzyme by Dafaglitapin (Dafa) has been shown to have therapeutic benefits in these conditions [16, 19, 24, 25]. Functionally, ALCAT1-mediated cardiolipin remodelling serves as a vital regulator of mitochondrial function. Li et al. found that ALCAT1-related abnormal remodelling of cardiolipin linked mitochondrial dysfunction to obesity [16]. Similar findings have also been confirmed in Parkinson’s disease [26]. However, whether ALCAT1-mediated cardiolipin remodelling is involved in hyperglycaemia-induced podocyte injury and the related molecular mechanisms remain unknown.

In the present study, we explored the role of ALCAT1 in cardiolipin remodelling and mitochondrial dysfunction in podocytes under diabetic conditions, and we also investigated the correlated molecular mechanisms to provide a theoretical basis for DKD.

## Materials and methods

### Human renal biopsy samples

DKD patient samples were obtained from the Division of Nephrology at Renmin Hospital of Wuhan University, China. Control paraneoplastic tissues were obtained from individuals who underwent nephrectomy for kidney cancer at the Department of Urology of the hospital and did not have diabetic nephropathy (DN) or other diseases. Prior to the experiment, patients provided informed consent. The study was approved by the Wuhan University Research Ethics Committee and conducted in accordance with the guidelines of Wuhan University. The investigation also adhered to the principles of the Helsinki Declaration.

### Animal studies

Male db/db and db/m mice were obtained from CAVENS Laboratory Animals (Jiangsu, China) at 7 weeks of age. At 8 weeks of age, the mice were intrarenally administered adeno-associated virus (AAV) (OBiO Technology, China) through intraparenchymal injections with titres of approximately 2.0 × 10^12^ viral genomes/mL [27, 28]. The AAV vector used was pAAV-U6-shRNA-CMV-EGFP-WPRE packaging shALCAT1 (ALCAT1-AAV) or a negative control (NC-AAV). The mice were randomly divided into six groups: in the first group, NC-AAV injection was administered db/m mice (*n* = 6). In the second group, ALCAT1-AAV was administered to db/m mice (*n* = 6). In the third group, db/db mice were treated with NC-AAV (*n* = 6). In the fourth group, mice with db/db backgrounds received ALCAT1-AAV injection (*n* = 6). In the fifth group, db/db mice received an intraperitoneal injection of 3 mg/kg/day SS-31 (TargetMol, USA), a specific cardiolipin oxidation inhibitor, for 12 weeks (*n* = 6). This dosage of SS-31 was found to be effective without adverse side effects in related studies [29, 30]. In the last group, mice were treated with ALCAT1-AAV and Compound C (MCE, USA, 0.2 mg/kg, once daily by intraperitoneal injection for 30 days). Compound C is a widely used selective ATP-competitive AMPK inhibitor [31]. Blood glucose and body weight were tested every two weeks, and the urinary albumin creatinine ratio (ACR) was examined monthly after injection. For blood glucose measurement, a blood collection needle was used to take blood from the tail vein of mice and measured it with a glucometer. Kidneys and blood samples were collected at 20 weeks of age for histological and biochemical analysis. All in vivo experiments were approved by the animal experimental ethics committee of Renmin Hospital of Wuhan University.

### Lipidomic analysis

At 20 weeks of age, kidney tissues were collected from mice, homogenized, and subjected to methanol treatment. After sample processing, detection was performed using ultra-high-performance liquid chromatography and high-resolution mass spectrometry. The mass spectrometry results were analyzed and processed using Lipid Search software (Thermo Fisher Scientific, USA).

### Cell culture and treatments

Conditionally immortalized human podocytes were kindly provided by Professor Moin A. Saleem (Academic Renal Unit, Southmead Hospital, Bristol, UK). The podocytes were cultured at 33 °C in RPMI 1640 (HyClone, USA) medium supplemented with 10% thermally inactivated foetal bovine serum (FBS; Gibco, USA), 100 U/mL penicillin‒streptomycin (Invitrogen, USA), and 1× insulin-transferrin-selenium (ITS; Invitrogen, USA). To induce differentiation, the podocytes were incubated at 37 °C in medium without ITS for 10–14 days, after which they were used in all experiments. To stimulate podocyte differentiation, cells were exposed to 30 mM high glucose for the required duration. For the SiRAN and plasmid transfection analysis, podocytes were transfected with siRNA-ALCAT1 (DesignGene, China) according to the manufacturer’s instructions. A mixture containing 10 nM siRNA-ALCAT1 or scrambled siRNA plus HiPerFect transfection reagent (Qiagen, Germany) was applied to cells seeded in 6-well plates under normal or HG-treated conditions for 24 h. Similarly, for the overexpression analysis, 2 µg of human ALCAT1 overexpression plasmid or pcDNA3.1 was transfected into podocytes for 24 h using Lipo3000 (Thermo Fisher Scientific, USA). For the SS-31 and Compound C experiment, podocytes were treated with 100 nM SS-31 (MedChemExpress, China) or 10 µM Compound C for 24 h [32, 33]. Each experiment was validated using three independent podocyte clones.

### Immunofluorescence analysis

The paraffin sections were first dewaxed and then subjected to antigen retrieval. The slides were blocked for 30 min with 5% bovine serum albumin (BSA) and then incubated overnight at 4 °C with anti-synaptopodin (1:100, Santa, USA) and anti-ALCAT1 (1:100, Novus, USA) antibodies. On the next day, a mixture of fluorescent secondary antibodies was applied to the slides and incubated at room temperature in darkness for 1 h. DAPI (Antgene, China) was used to stain the nuclei for 5 min. Cultured podocytes were fixed on cover slides in 4% paraformaldehyde at 4 °C for half an hour and then incubated with ALCAT1 antibody (1:100, Novus, USA) overnight at 4 °C. Finally, the nuclei were stained with DAPI (Antgene, China). Images were captured using a confocal microscope (Olympus, Japan).

### Immunohistochemistry analysis

Immunohistochemistry was used to determine the expression of ALCAT1 in kidney tissue. First, antigen retrieval was performed on dewaxed kidney paraffin sections. Then, the slides were blocked using 5% BSA and incubated with the primary antibody and subsequently the secondary antibody. DAB staining was applied, followed by haematoxylin staining. Finally, images were captured using an Olympus microscope (Japan). When conducting quantitative analysis, for patient specimens, 15 glomeruli were randomly selected in four different sections from each patient and the average values were used for analysis. And for mouse specimens, 30 glomeruli were randomly selected in five different sections per animal in each group for analysis.

### Pathologic analysis

To assess the pathological damage of the glomeruli, such as sclerosis and mesangial expansion, paraffin-embedded sections were deparaffinized and stained with haematoxylin-eosin (HE) and periodic acid-Schiff (PAS). The ultrastructure of renal tissues was examined and analysed by electron microscopy after fixing them with 2.5% glutaraldehyde.

### Western blotting

The sieving method was used to isolate glomeruli, as previously described [34]. Total protein was extracted from both cultured podocytes and isolated glomeruli using RIPA buffer containing a protease inhibitor cocktail (Sigma‒Aldrich, USA), PMSF (Beyotime, China), and a phosphatase inhibitor (Beyotime, China). After electrophoresis and electroblotting, the samples were incubated with primary and secondary antibodies and visualized on a chemiluminescence instrument (Bio-Rad, USA). The resulting bands were analysed using ImageJ. Western blotting was performed using the following antibodies: anti-ALCAT1 (1:1000, Invitrogen, USA), anti-β-actin (1:2000, Proteintech, China), anti-fission 1 (1:1000, FIS1; GeneTex, USA), anti-dynamin-related protein-1 (1:1000, DRP1; ImmunoWay, USA), anti-optic atrophy-1 (1:1000, OPA1; ImmunoWay, USA), anti-mitofusin2 (1:1000, MFN2; GeneTex, USA), anti-B-cell lymphoma-2 (1:1000, BCL2; Cell Signaling Technology, USA), anti-BCL2-Associated X (1:1000, BAX; Cell Signaling Technology, USA), anti-Caspase3 (1:1000, Cell Signaling Technology, USA), anti-PINK1 (1:1000, Novus, USA), anti-LC3B (1:1000, Abcam, USA), anti-P62 (1:1000, GeneTex, USA), anti-AMPK (1:1000, ImmunoWay, USA), anti-phospho-AMPK (1:1000, ImmunoWay, USA), and HRP-linked goat anti-rabbit/mouse IgG (1:10000, Antgene, China).

### MitoTracker Red Staining

To stain cells with MitoTracker Red (Invitrogen, USA), the following protocol was implemented [35]. Podocytes were incubated with a 50 nM MitoTracker Red working solution at 37 °C for 30 min, followed by staining with DAPI. The images were captured using fluorescence microscopy (Olympus, Japan).

### Measurement of mitochondrial functions

A series of experiments were performed to assess the generation of mitochondrial superoxide (MitoSOX), reactive oxygen species, mitochondrial membrane potential, and ATP production [36]. To evaluate the production of ROS in glomeruli and podocytes, DHE (Invitrogen, USA) and DCFH-DA (Beyotime, China) staining assays were used. Mitochondrial superoxide species production was assessed using the Red Mitochondrial Superoxide Indicator (Invitrogen, USA). Mitochondrial Membrane Potential (MMP) is the electrical potential difference across the inner mitochondrial membrane, essential for cellular energy production and various mitochondrial functions. And it was determined by staining with JC-1 (Beyotime, China), while the ATP content was measured using an ATP Determination Kit (Beyotime, China).

### Apoptosis assay

To detect podocyte apoptosis, a PE and 7-ADD double staining method with flow cytometry was used. The Annexin V Apoptosis Detection Kit I from BD Pharmingen (USA) was utilized. The manufacturer’s instructions were followed throughout the entire procedure.

### Statistical analyses

Statistical analysis was performed using GraphPad Prism 9. The results are presented as the mean ± standard deviation (SD). Differences in mean values were evaluated using Student’s t test or one-way ANOVA. A *P* value < 0.05 was considered to indicate statistical significance.

## Results

### ALCAT1 levels were increased in podocytes from the renal biopsies of DKD concomitant with mitochondrial abnormalities

**Fig. 1:**
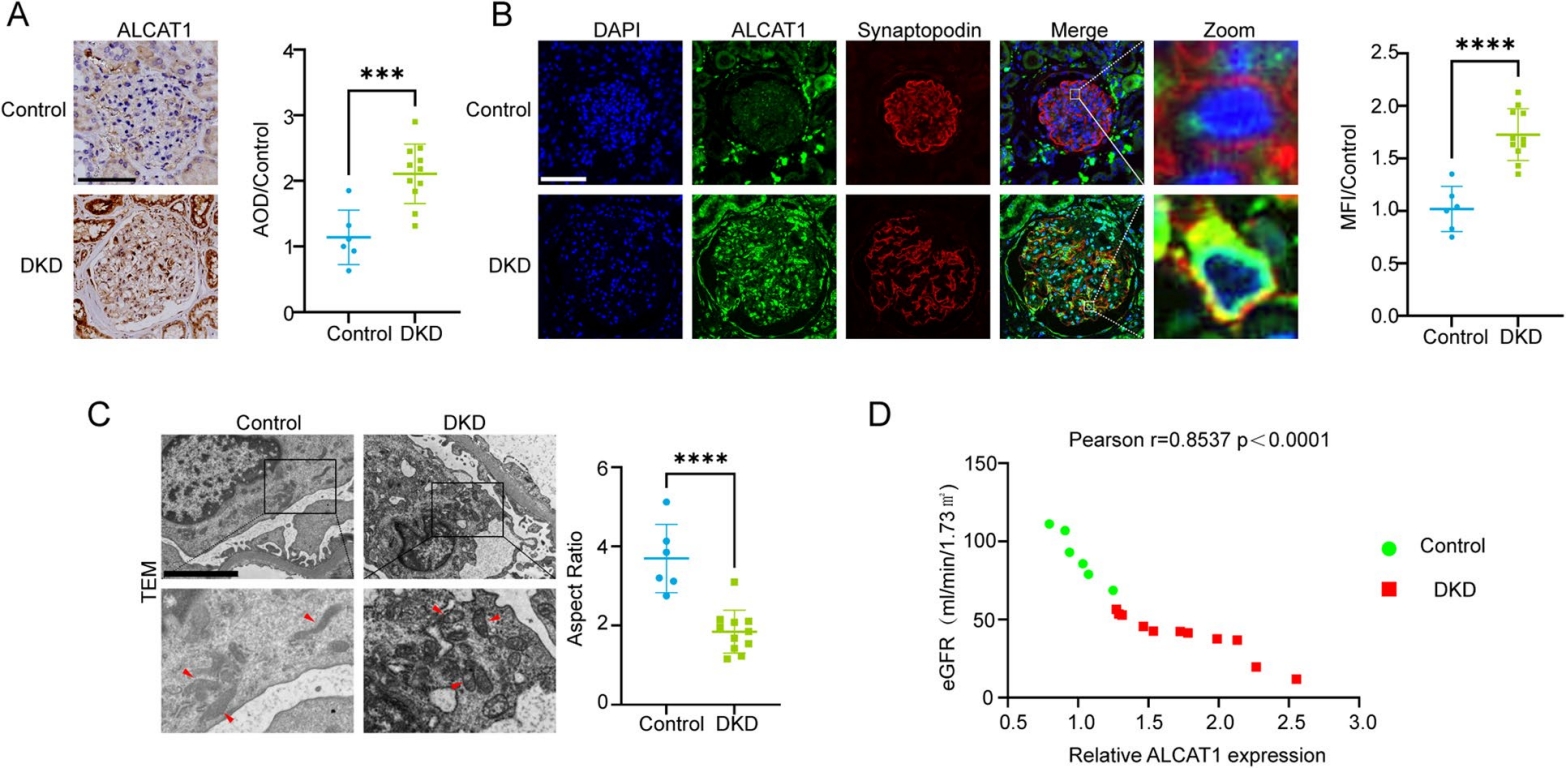
ALCAT1 levels were increased in glomerular podocytes along with mitochondrial injury in individuals with DKD. **A** Immunohistochemical staining and semiquantitative analysis of ALCAT1 in the glomeruli of patients with DKD (*n *= 11) and control participants (*n* = 6) (***p<0.001, scale bars: 80 μm). **B** Immunofluorescence double staining and semiquantitative analysis of ALCAT1 and synaptopodin in glomeruli from patients with DKD (*n *= 11) and control participants (*n *= 6) (****p<0.0001, scale bars: 80 μm). **C** Transmission electron micrography (TEM) results of podocyte foot processes, glomerular basement membrane (GBM) and mitochondrial morphology from patients with DKD (*n *= 11) and control participants (*n* = 6) (****p<0.0001, scale bars: 4 μm). **D** Correlation between ALCAT1 expression and eGFR (Control group: *n* = 6; DKD group: *n* = 11)

In an in vivo study, we aimed to investigate the impact of diabetes on ALCAT1 expression in glomeruli. We employed immunohistochemistry (IHC) to stain renal biopsies for ALCAT1. As shown in Fig. 1A, we observed a significant increase in ALCAT1 expression in the glomeruli of patients with DKD. To further assess ALCAT1 expression in podocytes of kidney tissue, we conducted fluorescence double staining of ALCAT1 and synaptopodin, a podocyte marker. The results indicated that ALCAT1 expression was elevated in podocytes of DKD patients compared to controls (Fig. 1B). Since podocytes are the key target of early damage in DKD, their injury and loss are closely linked to the disease. Via electron microscopy, we observed an increase in the thickness of the glomerular basement membrane (GBM) and fusion of the foot processes. Mitochondrial morphology was significantly abnormal, exhibiting mitochondrial swelling, cristae rupture, and mitochondrial fragmentation (Fig. 1C). We gathered data on the estimated glomerular filtration rate (eGFR) of these patients (Supplementary Table) and conducted immunohistochemistry analyses to determine the expression of ALCAT1 in their glomeruli. Our findings indicated a negative correlation between ALCAT1 expression and the glomerular filtration rate (Fig. 1D). Thus, we postulated that ALCAT1 upregulation might contribute to mitochondrial dysfunction and podocyte injury.

### ALCAT1 was upregulated in glomerular podocytes from db/db mice accompanied by mitochondrial abnormalities

**Fig. 2:**
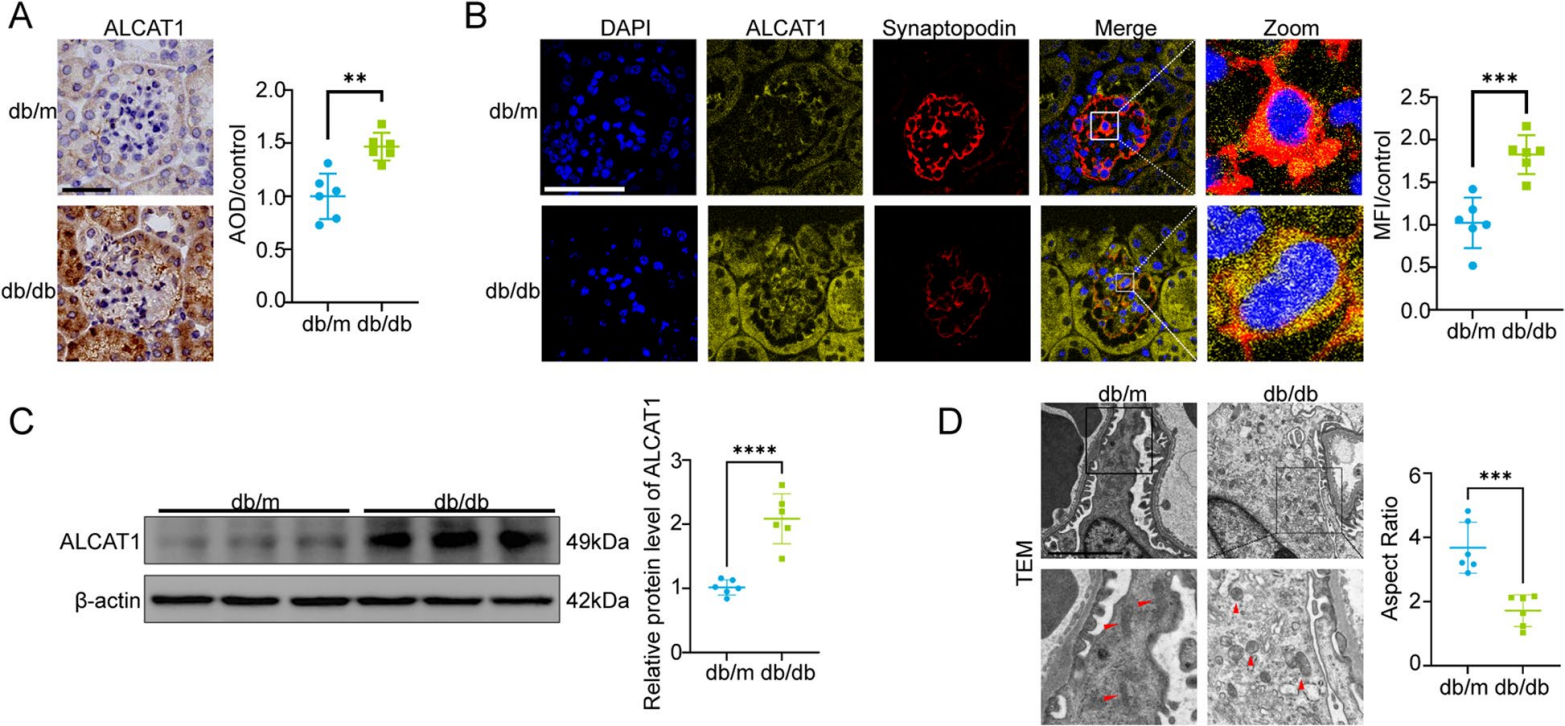
ALCAT1 was increased in the glomerular podocytes of db/db mice, accompanied by mitochondrial damage. **A** Immunohistochemical staining and semiquantitative analysis of ALCAT1 in glomeruli from each group (*n* = 6, ***p<0.001, scale bars: 40 μm). **B** Immunofluorescence double staining and semiquantitative analysis of ALCAT1 and synaptopodin in glomeruli in each group (*n* = 6, ***p<0.001, scale bars: 60 μm). **C** Western blots and relative expression of ALCAT1 (*n* = 6, ****p<0.0001). **D** Representative transmission electron micrographs of capillary loops and mitochondrial morphology in the glomeruli from each group (*n* = 6, ***p<0.001, scale bars: 2 μm)

To evaluate the effect of HG on ALCAT1 expression in podocytes from db/db mice, we first performed immunohistochemical staining for ALCAT1 and found an upregulation of ALCAT1 expression in the renal glomeruli of diabetic mice. To further investigate the expression of ALCAT1 in the podocytes of the renal glomeruli, we performed dual immunofluorescence staining. Synaptopodin and ALCAT1 double immunofluorescence staining revealed that ALCAT1 was upregulated in glomerular podocytes from the db/db group, consistent with our findings in DKD patients (Fig. 2A-B). Western blot results also confirmed a significant increase in ALCAT1 protein expression in db/db mice (Fig. 2C). Electron microscopy showed significant podocyte injury in db/db mice, mainly manifested as exacerbated diffuse foot processes, GBM thickening and mitochondrial morphological structural disorders (Fig. 2D). Taken together, these results demonstrated that mitochondrial dysfunction and podocyte injury might be attributed to the upregulation of ALCAT1.

### Knocking down ALCAT1 or inhibiting cardiolipin oxidation by SS-31 improved renal function and glomerular injury in diabetic mice

**Fig. 3:**
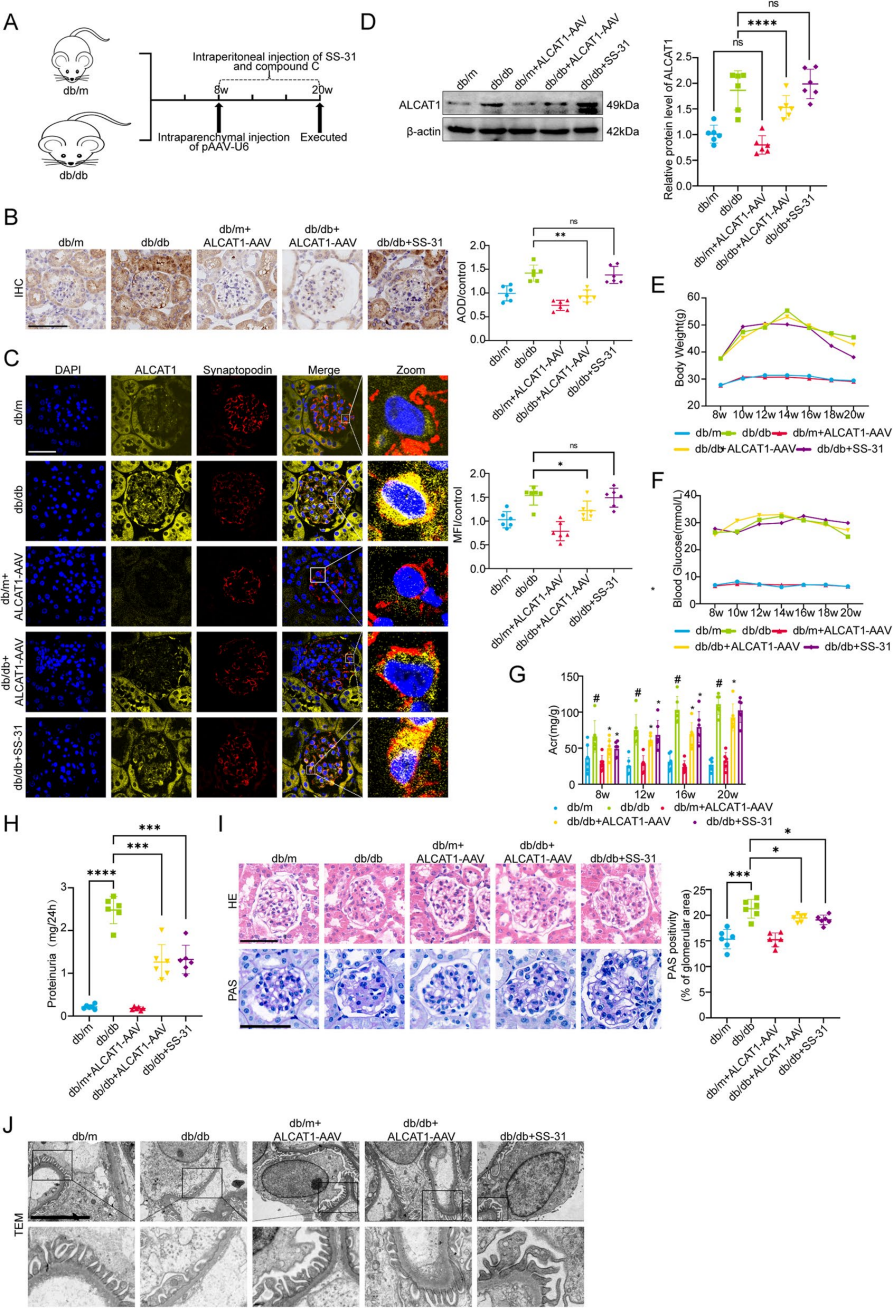
Knockdown of ALCAT1 or inhibition of cardiolipin oxidation ameliorated kidney injury. **A** Illustration of the Animal Model Construction Pathway. **B** Immunohistochemical staining and semiquantitative analysis of ALCAT1 in glomeruli from each group (*n* = 6, **p<0.01, ns *p*>0.05, scale bars: 40 μm). **C** Immunofluorescence double staining and semiquantitative analysis of ALCAT1 and synaptopodin in glomeruli in each group (*n* = 6, *p<0.05, ns *p*>0.05, scale bars: 40 μm). **D** ALCAT1 protein levels in each group evaluated by Western blot (*n* = 6, *p<0.05, ****p<0.0001, ns *p*>0.05). **E**-**G** Body weight, blood sugar and urinary albumin creatinine ratio (UACR) from each group (*n* = 6, *p<0.05, #*p*<0.0001). **H** Results of 24-hour urine protein quantification (*n* = 6, ***p<0.001, ****p<0.0001). **I** Pathological damage detection by HE and PAS staining of kidney sections and quantification of the extent of mesangial expansion (*n* = 6, *p<0.05, ***p<0.001, scale bars: 30 μm). **J** Ultrastructure of capillary loops according to transmission electron microscopy in each group (*n* = 6, scale bars: 6 μm)

To further investigate the role of ALCAT1 in podocytes in vivo, ALCAT1 expression was knocked down in db/db mice via intraparenchymal injections of adeno-associated virus. Additionally, 8-week-old db/db mice were intraperitoneally injected with SS-31 for three months for pharmacological inhibition of cardiolipin oxidation (Fig. 3A). The efficiency of virus infection was examined by evaluating the expression of the virus eGFP tag in frozen kidney sections (Supplementary Fig. 2). Immunohistochemical staining and Western blot analysis confirmed the successful knockdown of ALCAT1 in the mice injected with AAV, while SS-31 had little effect on ALCAT1 expression. Immunofluorescence results also showed similar results (Fig. 3B-D). Blood glucose and body weight in db/db mice with ALCAT1 knockdown or those subjected to SS-31 treatment were similar to those of db/db mice (Fig. 3E-F). However, we observed a marked decrease in the albumin-to-creatinine (ACR) ratio and 24-hour urine protein levels in db/db mice with ALCAT1 knockdown or those subjected to SS-31 treatment compared to the db/db group (Fig. 3G-H). Extracellular matrix accumulation and mesangial expansion in diabetic mice were ameliorated via ALCAT1 knockdown or cardiolipin oxidation inhibition, as shown by PAS and HE staining (Fig. 3I). Electron microscopy revealed that foot process fusion and glomerular basement membrane thickening were improved in db/db mice with ALCAT1 knockdown or those subjected to SS-31 treatment compared to the db/db group (Fig. 3J). These results collectively suggested the involvement of ALCAT1 and cardiolipin oxidation in the pathogenesis of DKD. ALCAT1 deficiency and the cardiolipin oxidation inhibitor SS-31 alleviated glomerular podocyte injury under diabetic conditions.

### ALCAT1 downregulation and SS-31 treatment improved cardiolipin remodelling and alleviated mitochondrial damage

**Fig. 4:**
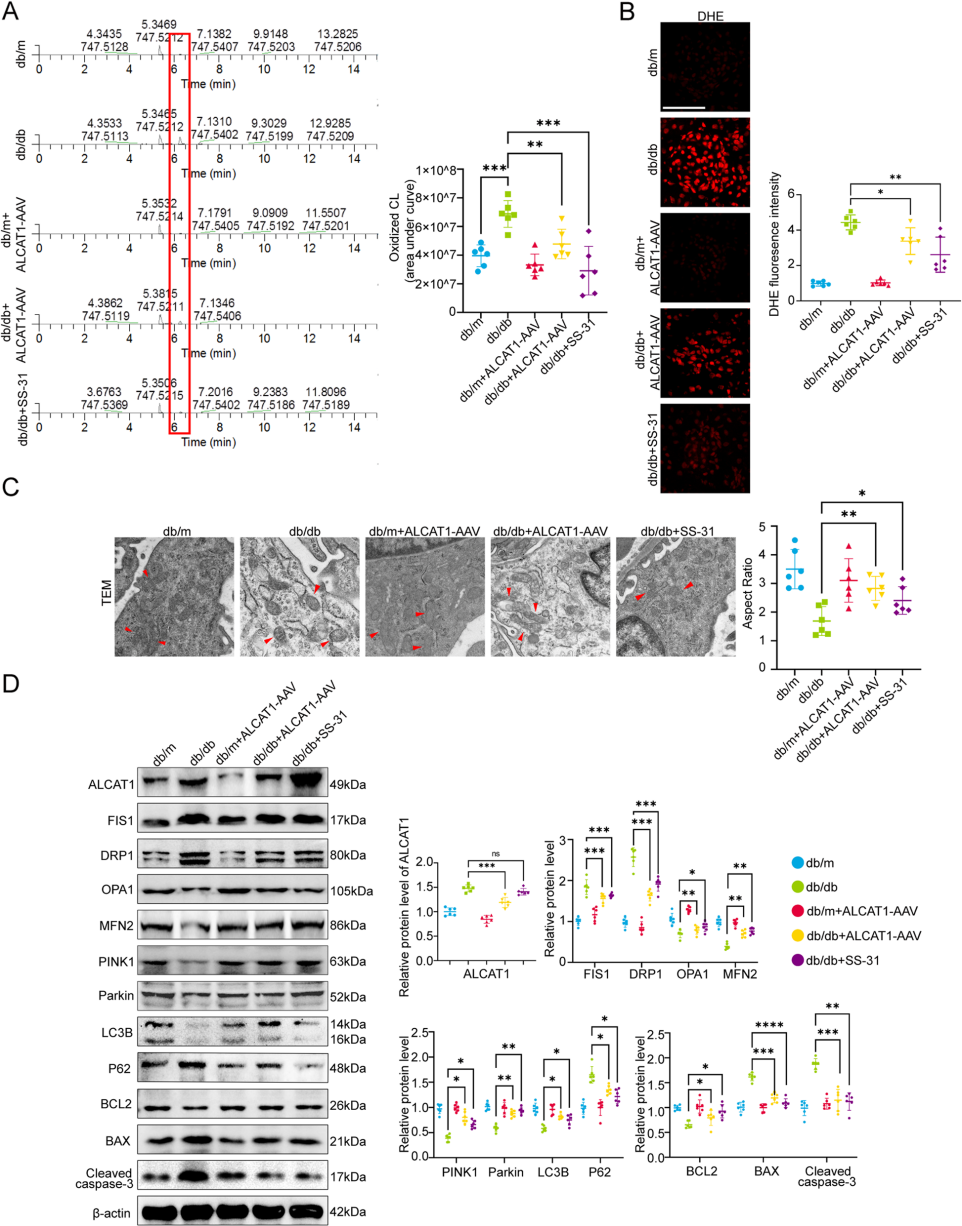
ALCAT1 inhibition or SS-31 treatment attenuated abnormal cardiolipin remodelling and podocyte mitochondrial damage in diabetic mice. **A** Relative content of oxidized cardiolipin in the renal cortex (*n* = 6, **p<0.01, ***p<0.001). **B** Micrographs and semiquantitative DHE staining in different groups (*n* = 6, *p<0.05, **p<0.01, scale bars: 30 μm). **C** Representative electron microscopy images of the ultrastructure of podocyte mitochondria from different groups (*n* = 6, *p<0.05, **p<0.01, scale bars: 1 μm). **D** Western blotting in the glomerulus of ALCAT1, mitochondrial fusion/fission-related proteins (FIS1, DRP1, OPA1, MFN2), autophagy-related proteins (PINK1, LC3B, P62), and apoptosis-related proteins (BCL2, BAX, cleaved caspase-3) (*n* = 6, *p<0.05, **p<0.01, ***p<0.001, ****p<0.0001, ns *p*>0.05)

It has been confirmed that ALCAT1 can exert a harmful effect on cells by regulating abnormal CL remodelling in myocardial cells and neuroblastoma cells, but its role in podocytes of the glomerulus is not yet clear. To further evaluate whether ALCAT1 downregulation affects abnormal renal CL remodelling, we used lipidomic analysis to monitor the content of oxidized CL. Lipid database analysis revealed that db/db mice displayed significant abnormal remodelling of cardiolipin compared to db/m mice, characterized primarily by an increase in oxidized cardiolipin. However, this abnormality could be prevented by treatment with SS-31 or ALCAT1 downregulation (Fig. 4A). ALCAT1 deficiency or SS-31 treatment reduced ROS production in the glomeruli of diabetic mice by using the fluorescent probe DHE (Fig. 4B), and db/db mice displayed excessive mitochondrial fragmentation, severe swelling, and mitochondrial cristae fracture or loss (Fig. 4C). These morphological and structural abnormalities of the mitochondria were improved by ALCAT1 knockdown or SS-31 administration in diabetic mice. We next analysed the expression of several key molecules associated with mitochondrial dynamics and mitophagy in glomeruli by Western blotting. Db/db mice displayed decreased expression of the mitochondrial fusion-related proteins OPA1 and MFN2 and increased expression of the mitochondrial fission-related proteins DRP1 and FIS1 in the glomeruli, which were reversed by SS-31 treatment or ALCAT1 downregulation. We are aware that the phosphorylation modifications of DRP1 are closely associated with its functionality. We conducted an analysis of pSer637-DRP1 and observed a significant reduction in phosphorylated DRP1 expression in db/db mice. Partial recovery was achieved following AAV-mediated knockdown of ALCAT1 or treatment with SS-31. This also suggests that in the diabetic state, DRP1 activation leads to mitochondrial dynamic abnormalities, resulting in mitochondrial fragmentation. ALCAT1 and cardiolipin are also implicated in this process. (Supplementary Fig. 3) We found that the autophagic regulators PINK1 and LC3B were downregulated, while P62 was upregulated in the db/db group, which indicated inhibition of mitochondrial autophagy. The expression of these major autophagic regulators was restored after intervention. The upregulation of BAX and cleaved caspase-3 and the downregulation of BCL2 expression in db/db mice indicated increased apoptosis resulting from diabetes. However, inhibition of cardiolipin oxidation or ALCAT1 knockdown partially alleviated apoptosis (Fig. 4D). Taken together, these observations suggested that cardiolipin remodelling contributed to oxidative stress and mitochondrial damage. This demonstrated that abnormal cardiolipin remodelling by ALCAT1 promoted podocyte mitochondrial malfunction and injury by influencing mitochondrial dynamics and mitophagy under diabetic conditions.

### ALCAT1 expression was upregulated along with abnormal cardiolipin remodelling and mitochondrial dysfunction in high glucose-treated podocytes

**Fig. 5:**
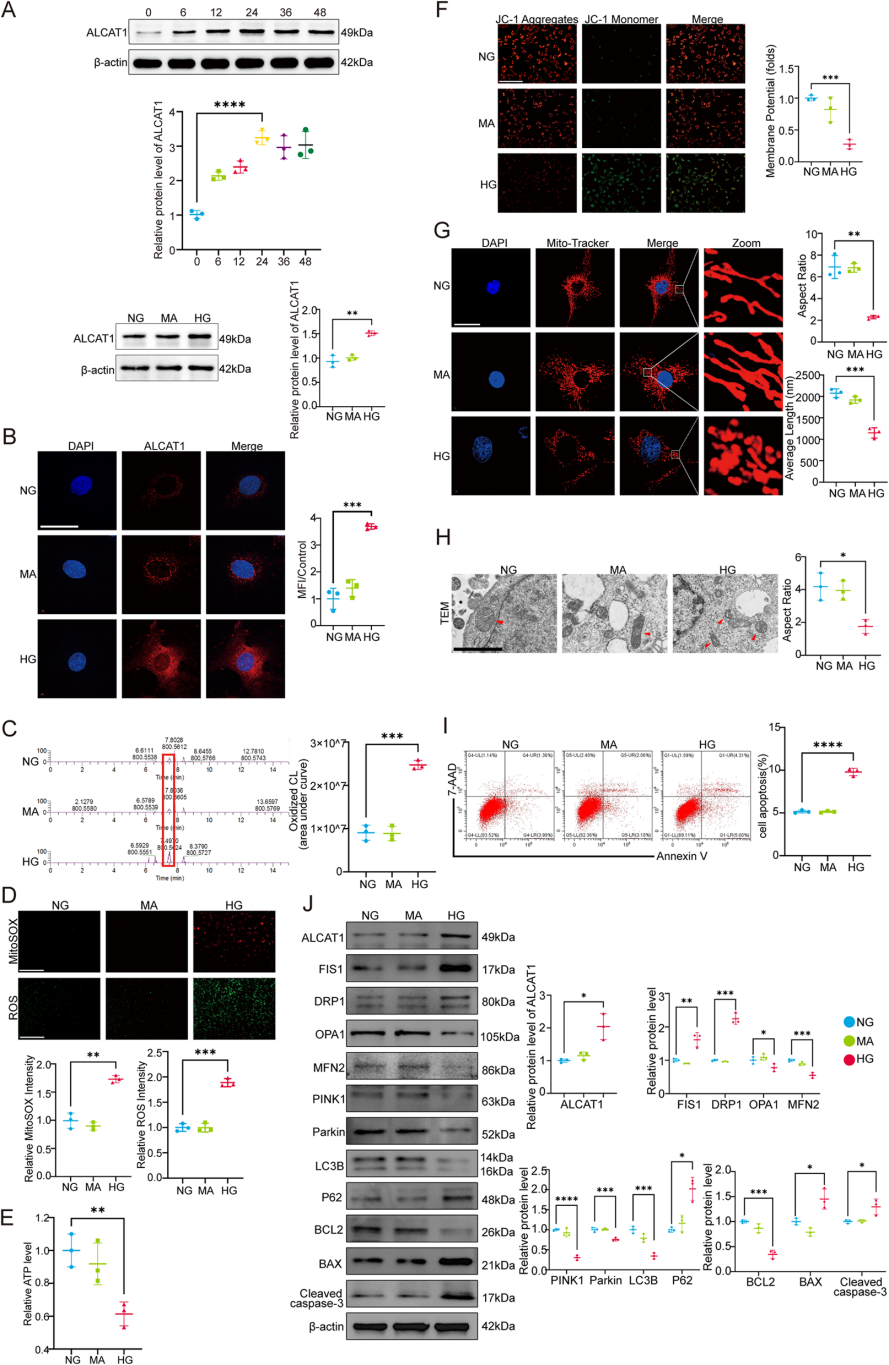
ALCAT1 was increased in HG-treated podocytes, accompanied by abnormal cardiolipin remodelling and mitochondrial dysfunction. **A **Western blotting was used to measure the variations in ALCAT1 levels in cultured podocytes subjected to glucose stimulation with time gradients (0, 6, 12, 24, 36, and 48 h) (*n* = 3, **p<0.01, ****p<0.0001). **B** Representative immunofluorescence staining for ALCAT1 (red) and semiquantitative analysis of these results in each group (*n* = 3, ***p<0.001, scale bars: 40 μm). **C** Relative content of oxidized cardiolipin (*n* = 3, ***p<0.001). **D** Cellular ROS and mitochondrial ROS production were assessed by DCFH-DA fluorescent probe and MitoSox Red fluorescence staining in different groups (*n* = 3, **p<0.01, ***p<0.001, scale bars: 200 μm). **E** Determination of relative ATP content in each group (*n* = 3, **p<0.01). **F** Relative quantification of mitochondrial membrane potential by JC-1 staining in podocytes (*n* = 3, ***p<0.001, scale bars: 200 μm). **G** Microscopy images of mitochondrial fission and fusion by MitoTracker Red staining and semiquantitative analysis of the average mitochondrial length and aspect ratio (*n* = 3, **p<0.01, ***p<0.001, scale bars: 40 μm). **H** TEM results of mitochondrial structure damage in podocytes (*n* = 3, *p<0.05, scale bars: 1 μm). **I** Apoptosis detection by flow cytometry in podocytes (*n* = 3, ****p<0.0001). **J** Western blotting of mitochondrial fusion/fission-related proteins (FIS1, DRP1, OPA1, MFN2), autophagy-related proteins (PINK1, LC3B, P62), and apoptosis-related proteins (BCL2, BAX, cleaved caspase-3) (*n* = 3, *p<0.05, **p<0.01, ***p<0.001, ****p<0.0001)

We conducted additional studies on cultured podocytes in vitro to determine whether high glucose (HG) could induce an increase in ALCAT1 and ultimately lead to cardiolipin remodelling. ALCAT1 protein levels in podocytes were increased in a time-dependent manner (0, 6, 12, 24, 36, and 48 h) under HG conditions (30 mM) (Fig. 5A). The expression of ALCAT1 in podocytes was not affected by mannitol (MA), which was used as a glucose (hypertonic) control. Immunofluorescence assays further revealed an increase in ALCAT1 expression in HG-stimulated podocytes (Fig. 5B). Cardiolipin oxidation was also exacerbated in podocytes treated with HG, which was consistent with the in vivo results (Fig. 5C). Cardiolipins play a crucial role in the function and structure of mitochondria. Mitochondrial function assays revealed that MMP and ATP production were decreased, while intracellular ROS and mitochondrial superoxide production were markedly induced in HG-treated podocytes (Fig. 5D-F). According to the MitoTracker Red staining results, the mitochondria of HG-exposed podocytes displayed fragmented, punctate, or round structures (Fig. 5G). Electron microscopy images showed significant damage to the mitochondrial structure of podocytes stimulated by HG, manifested mainly as mitochondrial swelling and mitochondrial ridge fractures (Fig. 5H). HG induced more severe apoptosis in podocytes based on flow cytometry analysis (Fig. 5I). Similar to the results in vivo, Western blot analysis also showed enhanced mitochondrial fission in HG-stimulated podocytes in vitro, as evidenced by activation of DRP1, reduced levels of pDRP1, and a decrease in fusion-associated proteins. In addition, Western blotting also confirmed suppressed autophagy and increased apoptosis under high-glucose conditions. (Fig. 5J) (Supplementary Fig. 4A). Therefore, HG-stimulated podocyte mitochondrial damage and injury might be attributed to abnormal cardiolipin remodelling mediated by ALCAT1.

### ALCAT1 knockdown ameliorated HG-induced anomalous cardiolipin remodelling and mitochondrial damage in podocytes in vitro

**Fig. 6:**
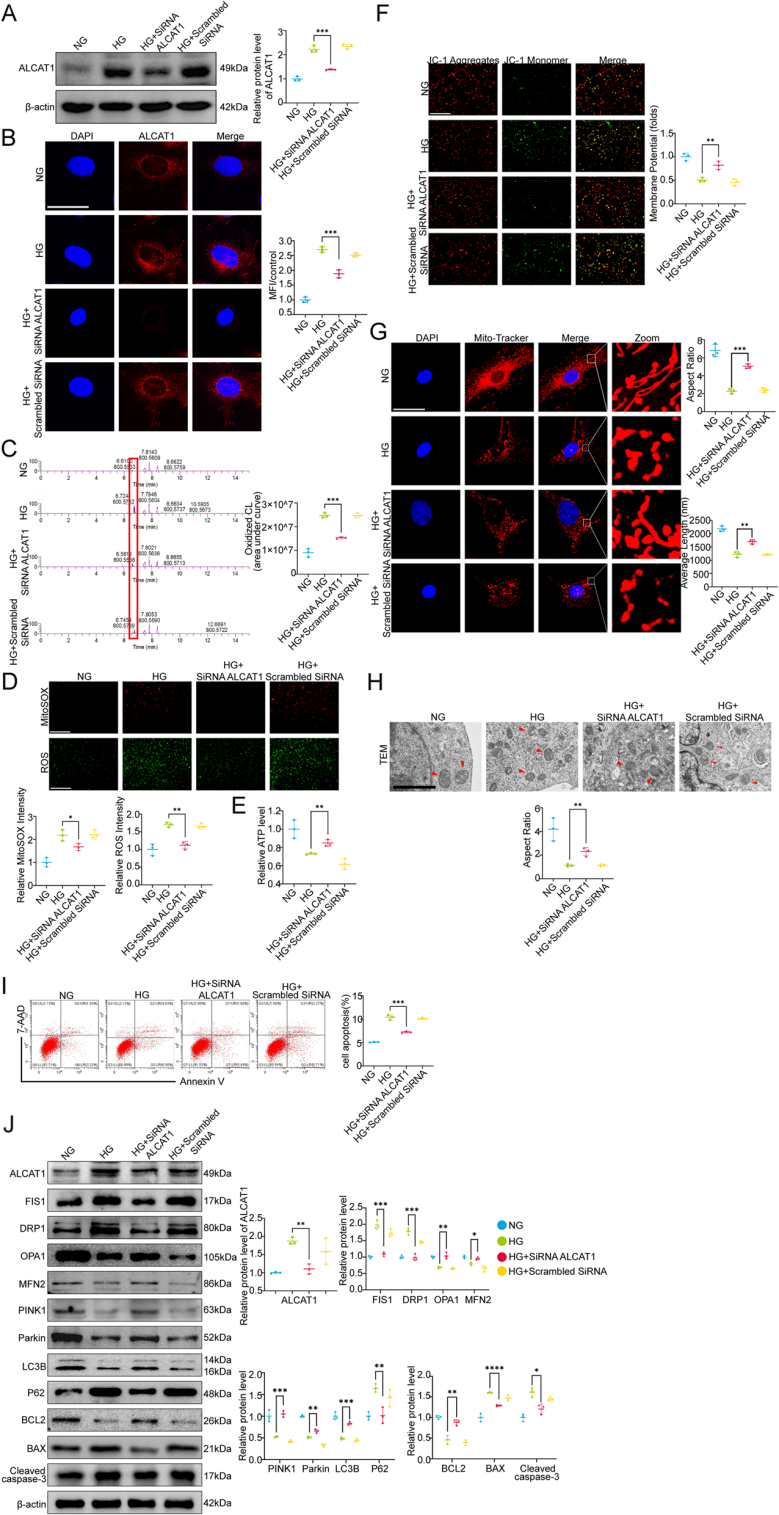
ALCAT1 deficiency improved abnormal cardiolipin remodelling and suppressed mitochondrial damage in high glucose-treated podocytes. **A** Verification of the knockdown effect of ALCAT1 siRNA by Western blotting (*n* = 3, ***p<0.001). **B** Immunofluorescence staining of ALCAT1 (red) in podocytes from each group (*n* = 3, ***p<0.001, scale bars: 40 μm). **C** Relative content of oxidized cardiolipin in podocytes measured by lipidomic analysis (*n* = 3, ***p<0.001). **D** Cellular ROS and mitochondrial ROS production were evaluated by DCFH-DA fluorescent probe and MitoSox Red fluorescence staining (*n* = 3, *p<0.05, **p<0.01, scale bars: 200 μm). **E** Relative ATP content and semiquantitative analysis of each group (*n* = 3, **p<0.01). **F** Relative quantification of mitochondrial membrane potential detected by JC-1 staining (*n* = 3, **p<0.01, scale bars: 200 μm). **G** Microscopy images of mitochondrial fission and fusion in podocytes and semiquantitative analysis of the average mitochondrial length and aspect ratio (*n* = 3, **p<0.01, ***p<0.001, scale bars: 60 μm). **H** Electron microscopy observation of the ultrastructure of mitochondria in podocytes (*n* = 3, **p<0.01, scale bars: 2 μm). **I** Apoptosis detection in each group by flow cytometry (*n* = 3, ***p<0.001). **J** Western blot analysis of mitochondrial fusion/fission-related proteins (FIS1, DRP1, OPA1, MFN2), autophagy-related proteins (PINK1, LC3B, P62), and apoptosis-related proteins (BCL2, BAX, cleaved caspase-3) (*n* = 3, *p<0.05, **p<0.01, ***p<0.001, ****p<0.0001)

Although ALCAT1 was involved in the regulation of cardiolipin remodelling in murine cardiomyocytes and motor neuron cells, its role in cardiolipin remodelling in podocytes was obscure. Thus, we conducted validation experiments to investigate whether ALCAT1 suppression could affect HG-induced abnormal podocyte cardiolipin remodelling and mitochondrial malfunction in vitro. To achieve this, we used siRNA transfection to effectively silence ALCAT1 expression, as confirmed by Western blot and immunofluorescence analyses (Fig. 6A-B). We performed lipidomic analysis to explore the effects of ALCAT1 inhibition on cardiolipin in HG-exposed podocytes. We observed a significant decrease in ox-CL levels in ALCAT1-silenced podocytes compared to those treated with high glucose alone (Fig. 6C). Furthermore, ALCAT1 silencing alleviated intracellular ROS production and mitochondrial oxidative stress in HG-treated podocytes and increased ATP content (Fig. 6D-F). The results revealed partial recovery of the mitochondrial morphology and structure in ALCAT1-silenced podocytes (Fig. 6G-H). In addition, ALCAT1 deficiency dramatically relieved HG-induced apoptosis in podocytes (Fig. 6I-J). Consistent with the results in diabetic mice, ALCAT1 knockdown reversed the key molecules involved in the regulation of mitochondrial fragmentation, mitophagy and apoptosis in HG-induced podocytes (Fig. 6J) (Supplementary Fig. 4B). In summary, these results suggested that the cytoprotective effect of ALCAT1 silencing on podocytes may be attributed to its ability to reduce abnormal remodelling of cardiolipin and protect mitochondria.

### Inhibition of cardiolipin oxidation by SS-31 blocked ALCAT1 overexpression-induced mitochondrial damage in HG-treated podocytes

**Fig. 7:**
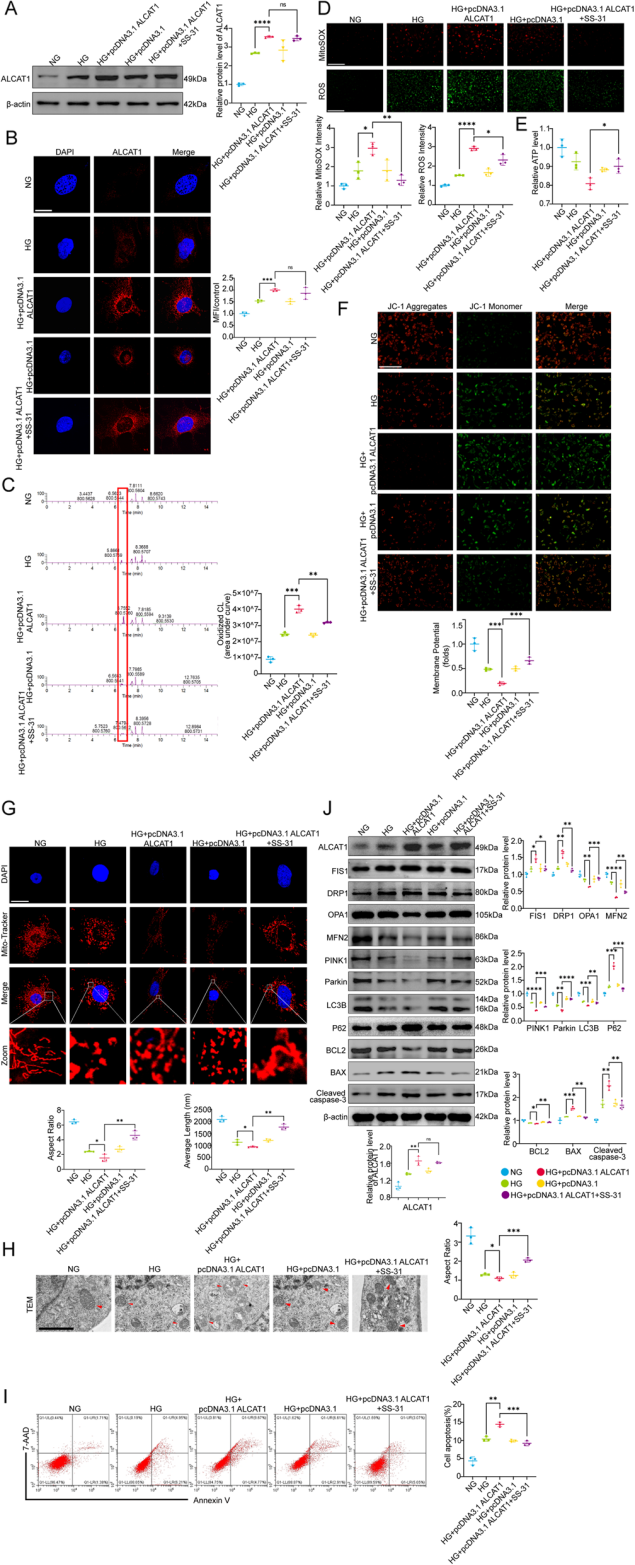
Inhibition of cardiolipin oxidation by SS-31 reversed ALCAT1 upregulation-mediated abnormal cardiolipin remodelling and mitochondrial damage in HG-induced podocytes. **A** The effects of overexpression plasmid transfection and SS-31 treatment on ALCAT1 expression were detected by Western blotting (*n* = 3, ****p<0.0001, ns *p*>0.05). **B** Immunofluorescence staining of ALCAT1 (red) in podocytes from each group (*n* = 3, ***p<0.001, ns *p*>0.05, scale bars: 20 μm). **C** Relative oxidized cardiolipin content in podocytes measured by lipidomic analysis (*n* = 3, **p<0.01, ***p<0.001). **D** Cellular ROS and mitochondrial ROS production were evaluated by DCFH-DA fluorescent probe and MitoSox Red fluorescence staining (*n* = 3, *p<0.05, **p<0.01, ****p<0.0001, scale bars: 200 μm). **E** Relative ATP content and semiquantitative analysis of each group (*n* = 3, *p<0.05). **F** Relative quantification of mitochondrial membrane potential detected by JC-1 staining (*n* = 3, ***p<0.001, scale bars: 200 μm). **G** Microscopy images of mitochondrial fission and fusion in podocytes by MitoTracker Red staining and semiquantitative analysis of the average mitochondrial length and aspect ratio (*n* = 3, *p<0.05, **p<0.01, scale bars: 40 μm). **H** Electron microscopy observation of the ultrastructure of mitochondria in podocytes (*n* = 3, *p<0.05, ***p<0.001, scale bars: 2 μm). **I** Apoptosis detection in each group by flow cytometry (*n* = 3, **p<0.01, ***p<0.001). **J** Western blot analysis of mitochondrial fusion/fission-related proteins (FIS1, DRP1, OPA1, MFN2), autophagy-related proteins (PINK1, LC3B, P62), and apoptosis-related proteins (BCL2, BAX, cleaved caspase-3) (*n* = 3, *p<0.05, **p<0.01, ***p<0.001, ****p<0.0001, ns *p*>0.05)

We next aimed to investigate whether ALCAT1 overexpression could worsen abnormal cardiolipin remodelling and mitochondrial damage in a high-glucose environment in vitro. To this end, podocytes were transfected with an ALCAT1 overexpression plasmid (pcDNA3.1-ALCAT1), followed by culture under HG conditions. The transfection efficiency was evaluated by Western blotting and immunofluorescence, as depicted in Fig. 7A-B. The overexpression of ALCAT1 exacerbated abnormal cardiolipin remodelling, as evidenced by a notable increase in the content of oxidized cardiolipin (Fig. 7C). Moreover, we observed that the damage to the mitochondrial function of podocytes was further aggravated, mainly manifested by increased generation of cellular and mitochondrial ROS and significantly reduced MMP and ATP content (Fig. 7D-F). Additionally, more fragmented mitochondria were observed in ALCAT1-overexpressing podocytes by MitoTracker staining and TEM (Fig. 7G-H). Flow cytometry analysis revealed that ALCAT1 overexpression triggered more podocyte apoptosis under HG stimulation (Fig. 7I). However, treatment with the specific cardiolipin oxidation inhibitor SS-31 partially alleviated all the injuries mentioned above. Consistent with our expectations, ALCAT1 upregulation aggravated the key molecules involved in mitochondrial fragmentation, mitophagy, and apoptosis in HG-induced podocytes (Fig. 7J) (Supplementary Fig. 4C). These results further suggested that ALCAT1-mediated abnormal cardiolipin remodelling provoked podocyte mitochondrial damage and cell injury under HG conditions.

### Abnormal cardiolipin remodelling induced by ALCAT1 caused mitochondrial dysfunction in podocytes through the AMPK signalling pathway

**Fig. 8:**
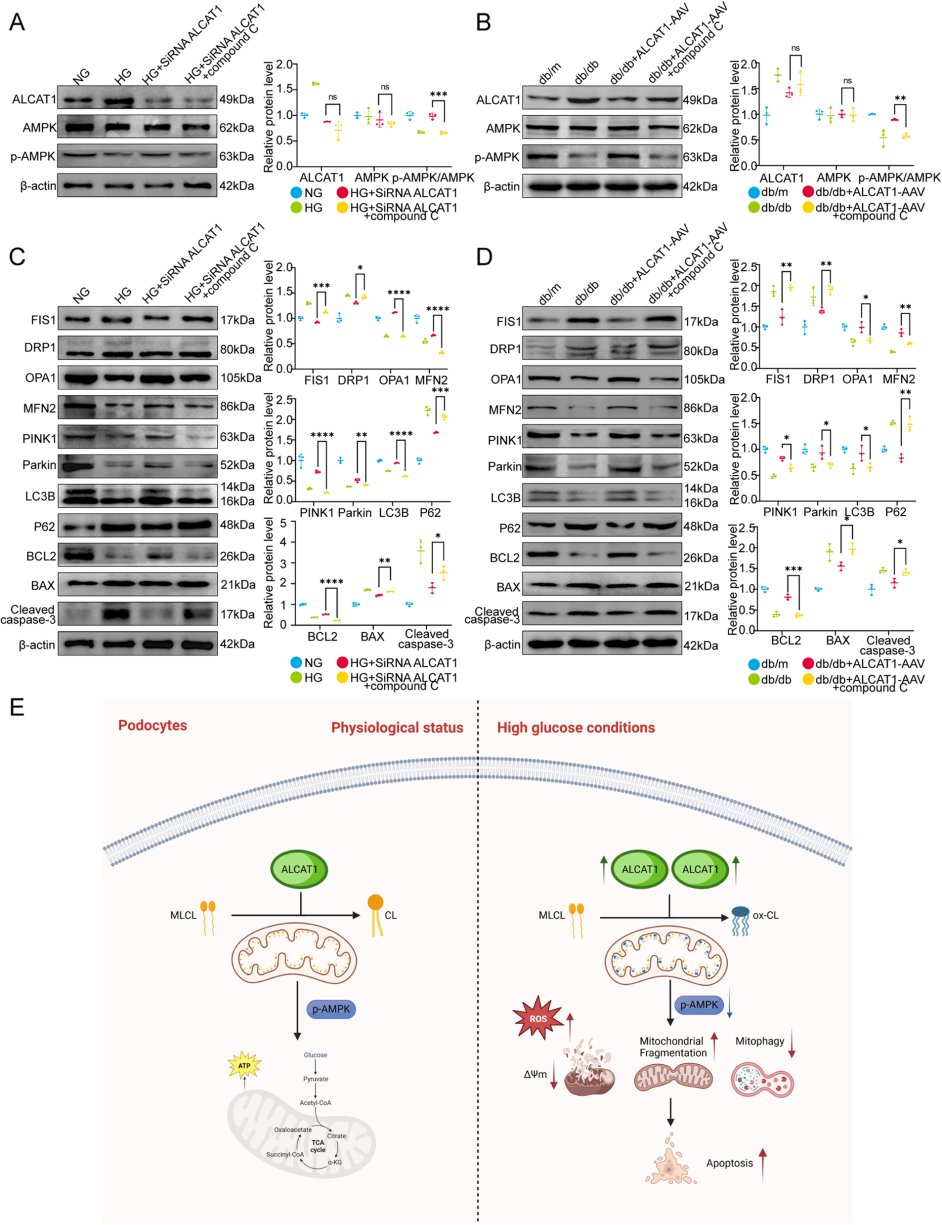
Aberrant cardiolipin remodelling mediated by ALCAT1 promoted mitochondrial malfunction in podocytes by deactivating the AMPK pathway. **A** Western blotting analysis of ALCAT1, AMPK and p-AMPK in vitro, as well as semi-quantitative of pAMPK/AMPK ratios (*n* = 3, ***p<0.001, ns *p*>0.05). **B** Western blotting analysis of ALCAT1, AMPK and p-AMPK in vivo, as well as semi-quantitative of pAMPK/AMPK ratios (*n* = 3, **p<0.01, ns *p*>0.05). **C** Western blot analysis of mitochondrial fusion/fission-related proteins (FIS1, DRP1, OPA1, MFN2), autophagy-related proteins (PINK1, LC3B, P62), and apoptosis-related proteins (BCL2, BAX, cleaved caspase-3) in vitro (*n* = 3, *p<0.05, **p<0.01, ***p<0.001, ****p<0.0001). **D** Western blot analysis of mitochondrial fusion/fission-related proteins (FIS1, DRP1, OPA1, MFN2), autophagy-related proteins (PINK1, LC3B, P62), and apoptosis-related proteins (BCL2, BAX, cleaved caspase-3) in vivo (*n* = 3, *p<0.05, **p<0.01, ***p<0.001). **E **Schematic of the molecular model proposed in this study

As previously mentioned, the disruption of mitochondrial homeostasis induced by high glucose may be associated with aberrant cardiolipin remodelling triggered by the upregulation of ALCAT1. There has been ample evidence to suggest that the AMPK signalling pathway plays a crucial role in regulating mitochondrial homeostasis [34, 37, 38]. To explore the molecular mechanism underlying these changes, we conducted a study to evaluate ALCAT1’s regulatory effects on AMPK. Our results showed that ALCAT1 silencing mitigated the HG-induced reduction in phospho-AMPK (Thr172) both in vivo and in vitro. Conversely, application of the AMPK inhibitor Compound C had the opposite effect. Compound C did not affect the expression of ALCAT1 (Fig. 8A-B). To further explore the role of the AMPK pathway in ALCAT1-induced mitochondrial malfunction in podocytes, we performed Western blot analysis to evaluate the key molecules involved in mitochondrial fragmentation, mitophagy and apoptosis in mice and cultured cells (Fig. 8C-D). Our findings showed that treatment with the AMPK inhibitor Compound C partially inhibited the cytoprotective effects mediated by ALCAT1 downregulation both in vivo and in vitro. Taken together, these results suggested that aberrant cardiolipin remodelling mediated by ALCAT1 promoted mitochondrial malfunction in podocytes by deactivating the AMPK pathway (Fig. 8E).

## Discussion

Podocyte injury and loss have emerged as crucial factors in the progression of DKD. In recent years, with the deepening of mitochondrial research, DKD has been recognized as a mitochondria-related disease. Zhou et al. revealed that high glucose levels lead to a reduction in mitochondrial autophagy in podocytes, consequently mediating podocyte apoptosis [39]. Ample evidence supports the claim that high glucose upregulates mitochondrial fission, swelling, and structural damage, as well as an increase in ROS production [8, 35, 38]. These findings strongly suggest that mitochondrial abnormalities may contribute to podocyte injury and DKD progression under high-glucose conditions. However, further studies are necessary to elucidate the underlying molecular mechanisms. A recent investigation has implicated ALCAT1, which is responsible for the pathological remodelling of cardiolipin with long-chain polyunsaturated fatty acids, in mitochondrial abnormalities in several diseases, including myocardial infarction, Parkinson’s disease, diet-induced obesity and other ageing-related diseases [15, 19, 22, 26]. In particular, in ageing-related diseases, ALCAT1 catalyses the pathological remodelling of cardiolipin, causing mitochondrial dysfunction [16, 25, 26]. Dafa, a highly potent ALCAT1-specific inhibitor, has been shown to restore mitochondrial function in myocardial infarction (MI) [19]. This evidence indicates that ALCAT1 is one of the key factors that regulates mitochondrial homeostasis. However, no relevant research has been conducted on podocytes in the context of diabetes. Herein, we demonstrated that ALCAT1-mediated abnormal cardiolipin remodelling may contribute to HG-induced mitochondrial damage and podocyte injury by inhibiting the AMPK pathway, which suggests that ALCAT1 might be a therapeutic target for DKD.

As the only lipid present on the mitochondrial membrane, CL plays a key role in maintaining normal cell function by supporting mitochondrial membrane structure and the activities of key enzymes involved in oxidative phosphorylation [15]. Therefore, appropriate cardiolipin composition and content are crucial for maintaining mitochondrial homeostasis and membrane integrity [40]. Research has shown that an increase in DHA-CL content due to abnormal remodelling is an important factor in causing left ventricular dysfunction in myocardial infarction [19]. The accumulation of harmful CL has also been demonstrated to exacerbate mitochondrial oxidative stress damage in mouse models of Parkinson’s disease [26]. In diet-induced obesity, abnormally remodelled CL leads to mitochondrial dysfunction, ROS production, and insulin resistance [16]. Among all phospholipids, CL contains high amounts of polyunsaturated fatty acids and resides exclusively within the mitochondrial membrane. The mitochondrial membrane is the main site of ROS production, which causes CL to be easily oxidized by ROS in DKD. Furthermore, a recent study reported that ox-CL was significantly increased in diabetic podocytes, accompanied by mitochondrial dysfunction [23]. Elamipretide (SS-31) has been demonstrated to protect db/db mice against the progression of DKD by inhibiting the oxidation of CL [41]. As a result of the accumulation of oxidized cardiolipin, podocytes in DKD may be susceptible to oxidative damage caused by mitochondrial oxidative stress. Our experimental results also confirm this conclusion. The lipidomic analysis of the renal cortex from db/db mice showed a substantial increase in oxidized cardiolipin, with a parent base peak between m/z values of 740–800 [23]. To further investigate the cause, we intervened with the expression of ALCAT1 both in vitro and in vivo and used the specific inhibitor SS-31 for targeted pharmacological inhibition of cardiolipin oxidation. The results showed that reducing ALCAT1 or inhibiting cardiolipin oxidation with SS-31 not only significantly decreased the content of ox-CL but also resulted in a significant improvement in mitochondrial function in podocytes.

As a mitochondria-targeted drug, SS-31 exerts a significant effect by binding with cardiolipin. SS-31 can inhibit cardiolipin peroxidation, participate in cardiolipin remodelling, restore cardiolipin levels, regulate the fusion of immature or mature long-chain cardiolipin with mitochondria, thus protecting the mitochondria. Studies have shown that SS-31 can improve oxidative stress and apoptosis by reducing the level of ROS in cells. In addition, SS-31 optimizes electron transfer and ATP synthesis, prevents the opening of the mitochondrial permeability transition pore (mPTP), inhibits mitochondrial swelling, reduces cytochrome C release, and prevents calcium overload [42]. Literature also suggests that SS-31 not only protects mitochondria but also restores damaged mitochondria in disease states [43]. So, we employed SS-31 to validate and explore the cardiolipin peroxidation induced by ALCAT1. Our findings also indicate that SS-31 can indeed alleviate the abnormal cardiolipin remodelling and oxidative cardiolipin increase caused by ALCAT1 overexpression [42–45].

Multiple mitophagy processes also require CL. When CL is oxidized, it is externalized to the mitochondrial surface, and ALCAT1 remodels it at the mitochondria-associated membrane (MAM), which acts as a key recognition signal for mitophagy [14, 46]. It has also been proposed in the literature that following mitochondrial damage, cardiolipin is externalised to the outer mitochondrial membrane where it can be recognised by LC3 proteins thereby initiating mitochondrial autophagy to remove damaged mitochondria. In particular, oxidised cardiolipin can be specifically recognised by LC3A [47].The degradation of defective mitochondria is an essential process to maintain cellular homeostasis [48]. However, there is evidence of defective mitophagy in type 2 diabetic mice, such that damaged mitochondria cannot be cleared in a timely manner [49]. In addition, cardiolipin is the most readily oxidized mitochondrial lipid, and its oxidation leads to the release of cytochrome c from mitochondrial intermembrane space, ultimately leading to apoptosis [21, 23, 50]. These data also supported our findings that in a high-glucose environment, the accumulation of ox-CL leads to inhibition of mitochondrial autophagy, triggering a cell death cascade. When we reduced the generation of oxidized cardiolipin by knocking down ALCAT1 or using SS-31, the structure of podocyte mitochondria was more stable, and the indicators related to mitophagy and apoptosis were significantly improved.

Glomerular disease is associated with mitochondrial fragmentation in podocytes [46, 51]. Our studies have demonstrated that mitochondrial dynamic homeostasis in podocytes plays a crucial role in the progression of DKD and hypertensive nephropathy [52]. However, the underlying causes are not yet fully understood. Mitochondrial fusion allows the functional complementation of partially dysfunctional mitochondria [14, 53]. In contrast, damaged mitochondria are eliminated by mitophagy after being isolated by fission [54]. During this process, multiple enzymes involved in mitochondrial dynamics require cardiolipin for their activity [14, 26, 37]. Previous studies have reported that in Parkinson’s disease, the abnormal restructuring of cardiolipin caused by ALCAT1 could alter the expression of proteins related to mitochondrial dynamics [26]. In our studies, we also observed that ALCAT1 upregulation led to abnormal accumulation of ox-CL in podocytes, resulting in decreased expression of fusion-related proteins (OPA1 and MFN2) and increased expression of fission-related proteins (DRP1 and FIS1). We also observed excessive mitochondrial fission by electron microscopy. However, this process could be mitigated through ALCAT1 downregulation or SS-31 treatment. Thus, we believe that attenuating the abnormal remodelling of cardiolipin by ALCAT1 knockdown or pharmacological inhibition of cardiolipin oxidation could alleviate mitochondrial dynamic damage.

Phosphorylation of Thr172 on the AMPK α subunit is essential for maintaining AMPK activity in renal tissue, which is crucial for energy metabolism and mitophagy regulation in podocyte mitochondria, as demonstrated in previous studies [38, 55–57]. The AMPK pathway is a critical regulatory factor in lipid metabolism within renal and other tissue cells. Studies have indicated that in high-fat diet-induced glomerular disease, the pAMPK/AMPK ratio significantly decreases, and treatment with SS-31 can improve this situation. The research also demonstrates that SS-31 does not impact systemic metabolic parameters such as body weight and blood glucose, nor does it directly activate AMPK. However, it is capable of restoring renal AMPK activity, thereby preventing the loss of glomerular endothelial cells and podocytes, and ultimately averting glomerular sclerosis [29]. In our study, AMPK activity was significantly inhibited in diabetic mice and HG-treated podocytes but restored upon ALCAT1 knockdown. To further verify the regulatory relationship between ALCAT1 and the AMPK signalling pathway, we treated ALCAT1 knockdown animals with Compound C, an AMPK signalling inhibitor. As anticipated, Compound C markedly inhibited the expression of p-AMPK but had no effects on ALCAT1 expression. Compound C treatment partly reversed the protective effects of ALCAT1 knockdown on podocyte mitochondria. In addition, we also overexpressed ALCAT in vitro cultured podocytes and treated them with an AMPK activator (AICAR,2mM,24 h). We observed that AICAR had no significant effect on ALCAT1 and AMPK expression, but it significantly restored pAMPK levels, leading to improvements in various mitochondrial damage indicators (Supplementary Fig. 5). Therefore, our findings suggested that ALCAT1 induced podocyte mitochondrial malfunction through the AMPK pathway. Some studies have reported that the knockout of AMPK may affect the expression of cardiolipin-related synthetic enzymes by possibly influencing the PGC-1α/ERRα axis, and this may be related to the role of AMPK in basal conditions [58]. In our research, the intervention with ALCAT1 did not affect the overall expression of AMPK but did impact the phosphorylated expression of AMPK. Further investigation is needed to determine whether the reduction in pAMPK alone affects the process of cardiolipin biosynthesis.

Thus, we speculated that ALCAT1 contributed to the pathogenesis of DKD through the following mechanisms: in pathological conditions, the abnormal overexpression of ALCAT1, due to its poor substrate selectivity, results in abnormal cardiolipin remodelling. Moreover, cardiolipin is located in the inner mitochondrial membrane, the site of ROS production. And it is rich in unsaturated fatty chains, especially cardiolipin which is abnormally reconstructed by very long-chain polyunsaturated fatty acids. For these reasons, it is highly susceptible to oxidation, mainly realized as the increase of oxidized cardiolipin. This cascade of events intensifies oxidative stress, with the increased ROS further affecting the AMPK signalling pathway, ultimately leading to mitochondrial structural damage and cell apoptosis (Fig. 8E). In summary, our findings suggested that ALCAT1-mediated abnormal cardiolipin remodelling promoted mitochondrial injury in podocytes in DKD by inhibiting the AMPK pathway. We also demonstrated that inhibiting ALCAT1 or cardiolipin peroxidation may represent a promising therapeutic approach for treating DKD patients.

There are several limitations to this study that need to be addressed in further investigations. First, our study only investigated the role of ALCAT1 in male diabetic mice, and it remains unclear whether ALCAT1 functions similarly in female mice. Second, we only used AAV to downregulate ALCAT1 expression in db/db mice, and further studies using overexpression or knockout mice are needed to confirm our findings. Finally, while our findings are promising, more studies are needed to determine whether targeting ALCAT1 can be applied clinically or at least in nonhuman primate models.

### Supplementary Information

**Additional file 1.**

**Additional file 2.**

** Additional file 3.**

## Data Availability

The datasets generated and analysed during the current study are accessible from the corresponding author upon reasonable request.

## Abbreviations

AAV: Adeno-associated virus
ALCAT1: Acyl-coenzyme A: lyso-cardiolipin acyltransferase-1
CL: Cardiolipin
DKD: Diabetic kidney disease
DN: Diabetic nephropathy
HE: Haematoxylin-eosin
HG: High glucose
MitoSOX: Mitochondrial superoxide
MMP: Mitochondrial membrane potential
ox-CL: Oxidized cardiolipin
PAS: Periodic acid-Schiff
ROS: Reactive oxygen species
TEM: Transmission electron microscope
