# Supplementary material for: ALCAT1-mediated abnormal cardiolipin remodelling promotes mitochondrial injury in podocytes in diabetic kidney disease
Source: Cell Commun Signal. 2024 Jan 10;22:26. doi: 10.1186/s12964-023-01399-4 (PMC10777643; doi:10.1186/s12964-023-01399-4)
Supplement: Supplementary file 2 — Additional file 1. [file 12964_2023_1399_MOESM1_ESM.docx]

**ONLINE SUPPLEMENT**

**Materials and methods**

**Anesthesia methods and specimen collection**

When performing intrarenal injections in mice, we utilized the Rodent Anesthesia Machine with isoflurane anesthesia. We induced anesthesia at a concentration of 3% to 4% isoflurane and maintained it at 1% to 1.5% isoflurane until the mice were fully anesthetized before proceeding with the surgery. Regarding the urine collection method, starting from 8-week-old mice, we collected urine samples monthly. Mice were placed in metabolic cages, and urine collection cups were sealed with mineral oil to prevent urine evaporation. After 24 hours, we collected the urine from the cups, recorded the volume, and then centrifuged it. We discarded the upper layer of oil and the bottom layer containing impurities, and used the clear middle layer of urine for subsequent creatinine (Acr) measurements. Regarding the blood collection method, at 20 weeks of age, mice were euthanized, and blood samples were collected promptly using cardiac puncture. After centrifugation to separate the layers, serum was collected for subsequent measurements of creatinine and other indicators. And for blood glucose measurement, a blood collection needle was used to take blood from the tail vein of mice and measured it with a glucometer.

**Supplementary Fig.1** ALCAT1 was increased in the renal tubule of db/db mice and HK2 cells under HG condition cultured in vitro. (A)Immunohistochemical staining and semi-quantitative of ALCAT1 in tubule from each group of mice(n=6, ***p＜0.001, scale bars: 40μm). (B)The cultured HK2 were stimulated with HG (30mM, 24h). Typical immunofluorescence staining for ALCAT1(green) and semi-quantitative analysis of these results in each group(n=3, **p＜0.01, scale bars: 40μm). (C)Western blots analysis and quantitative data showing ALCAT1 expression in cultured HK2 cells under HG conditions (n=3, **p＜0.01).


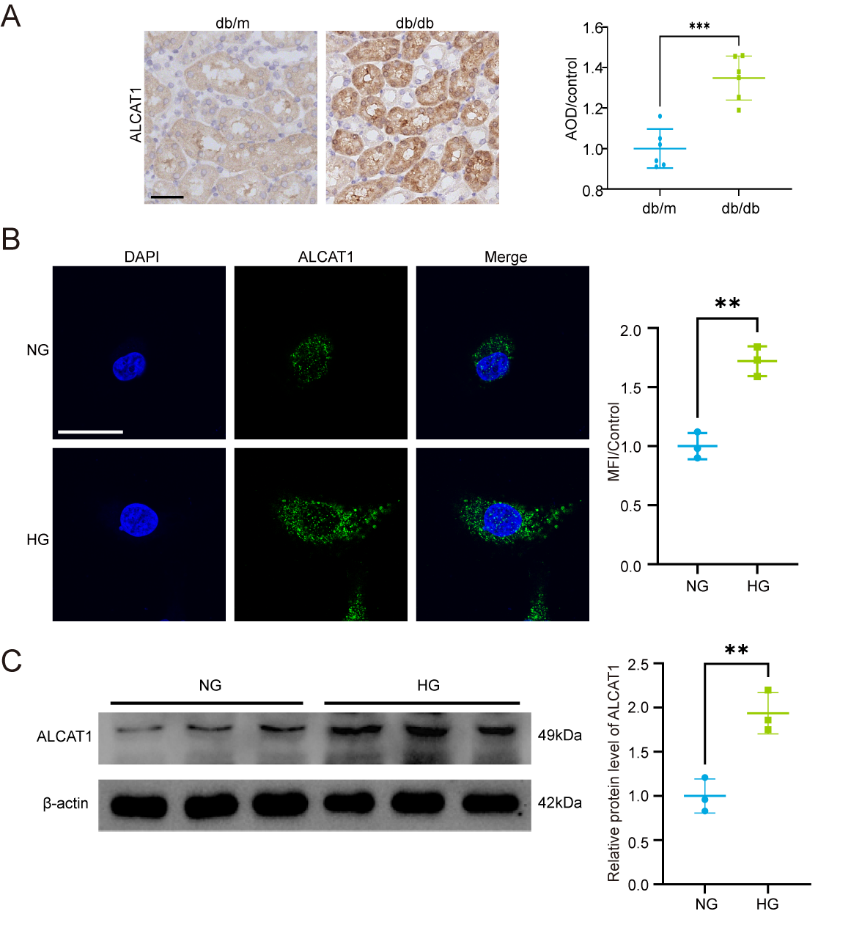


**Supplementary Fig.2** After injecting mice with adenovirus or SS-31, frozen sections were obtained and subjected to DAPI staining to observe eGFP fluorescence expression, confirming the efficiency of viral infection. Detection of the impact of SS-31 on eGFP fluorescence in mice (n=3, scale bars: 200μm).


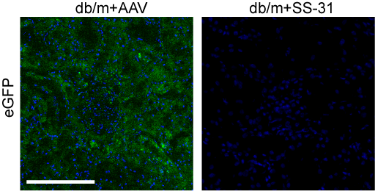


**Supplementary Fig.3** Western blotting analysis of pSer637-DRP1 in vivo, as well as semi-quantitative of pSer637-DRP1 (n=6, **p＜0.01, ***p＜0.001, ****p＜0.0001).


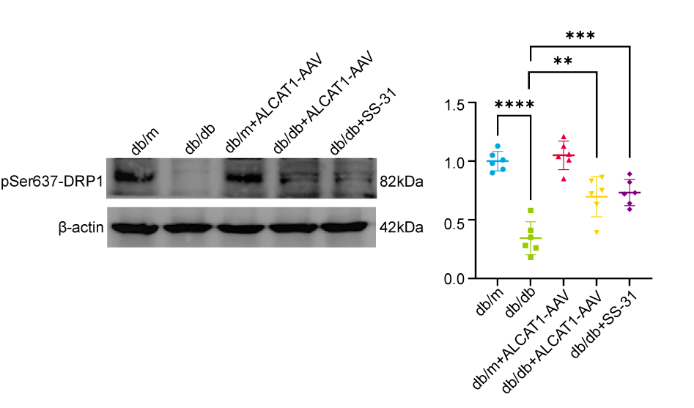


**Supplementary Fig.4** We supplemented Western blots to assess the effect of ALCAT1 siRNA, ALCAT1 plasmid and SS-31 on pSer637-DRP1 expression in HG-cultured podocytes. Western blotting analysis of pSer637-DRP1 in vitro, as well as semi-quantitative of pSer637-DRP1 (n=3, *p＜0.05, **p＜0.01).


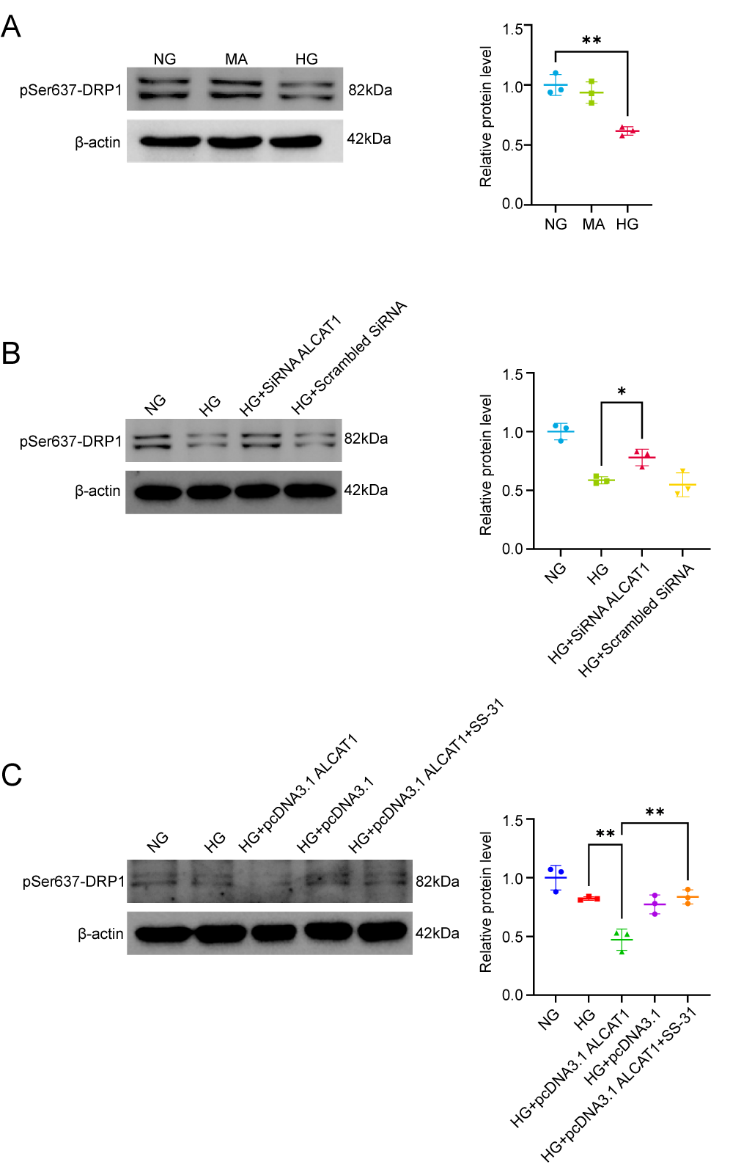


**Supplementary Fig.5** An AMPK agonist (AICAR, 2mM, 24h) was applied in ALCAT1 overexpressed podocytes in vitro to validate the relationship between ALCAT1 and AMPK. (A)Western blotting analysis of ALCAT1, AMPK and p-AMPK in vitro, as well as semi-quantitative of pAMPK/AMPK ratios. (n=3, ***p＜0.001, ns p＞0.05) (B)Western blot analysis of mitochondrial fusion/fission-related proteins (FIS1, DRP1, OPA1, MFN2), autophagy-related proteins (PINK1, LC3B, P62), and apoptosis-related proteins (BCL2, BAX, cleaved caspase-3) in vitro (n=3, *p＜0.05, **p＜0.01, ***p＜0.001, ****p＜0.0001).


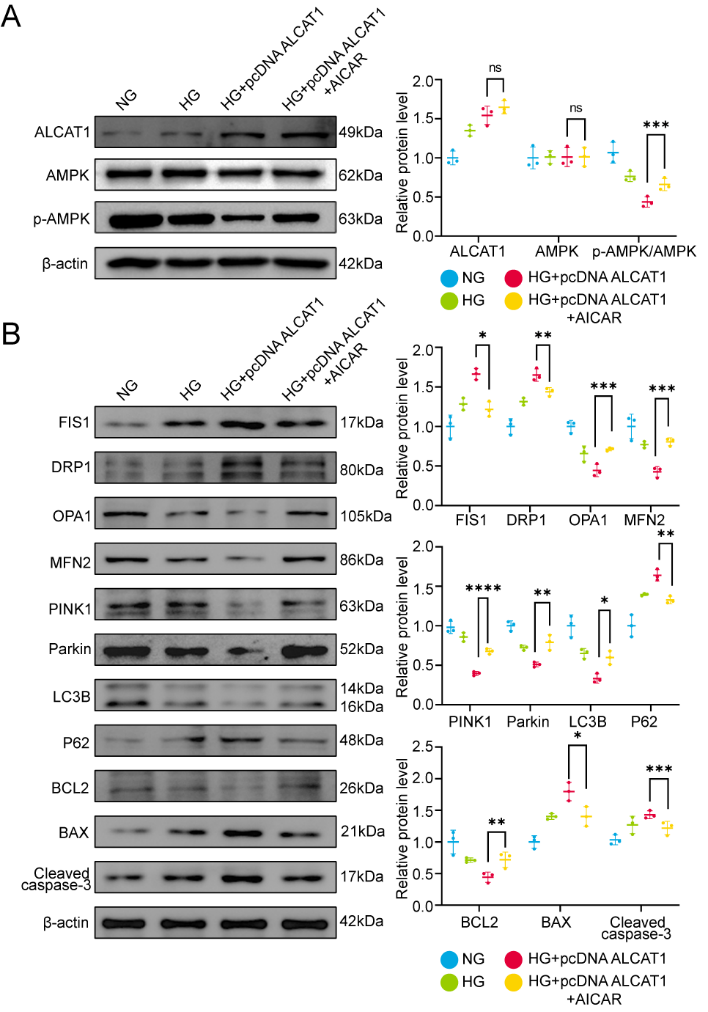


**Supplementary Table：**Clinical characteristics levels in control subjects and patients with DN. SCr, serum creatinine; yr, year; F, female; M, male; BUN, blood urea nitrogen; eGFR, estimated glomerular filtration Rate; FBG, fasting blood glucose; HbA1c, hemoglobin A1c; CHOL, total cholesterol; TG, triglycerides; NA, not applicable/not available.
