# Supplementary figures and images for: ALCAT1-mediated abnormal cardiolipin remodelling promotes mitochondrial injury in podocytes in diabetic kidney disease
Source: Cell Commun Signal. 2024 Jan 10;22:26. doi: 10.1186/s12964-023-01399-4 (PMC10777643; doi:10.1186/s12964-023-01399-4)

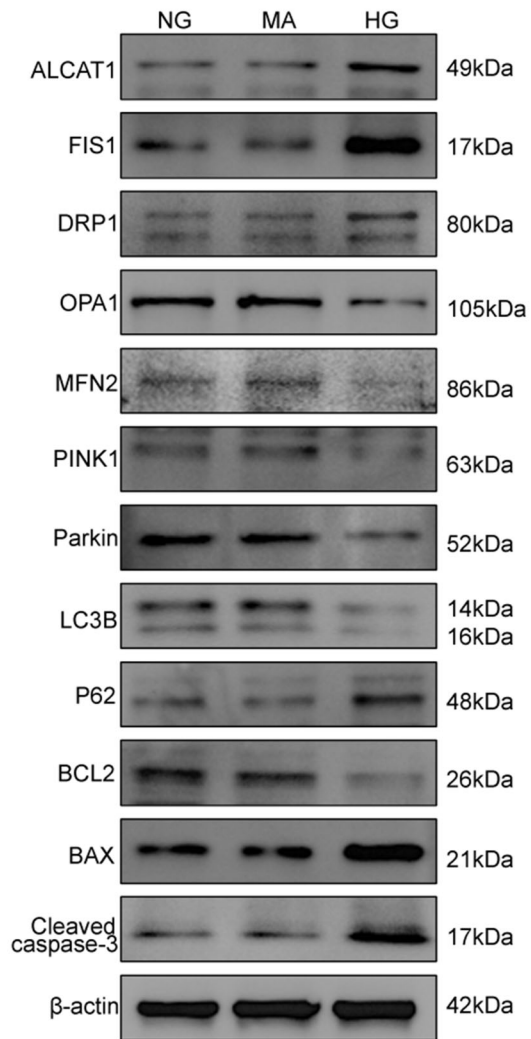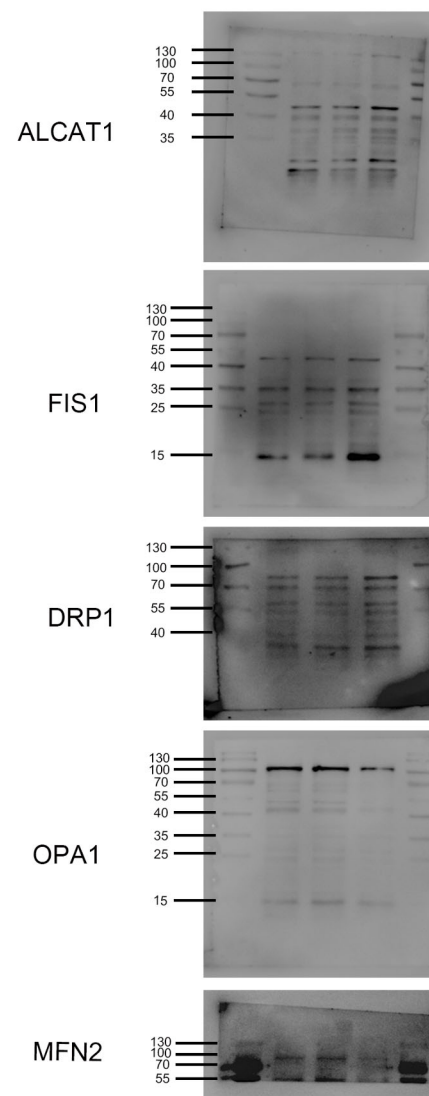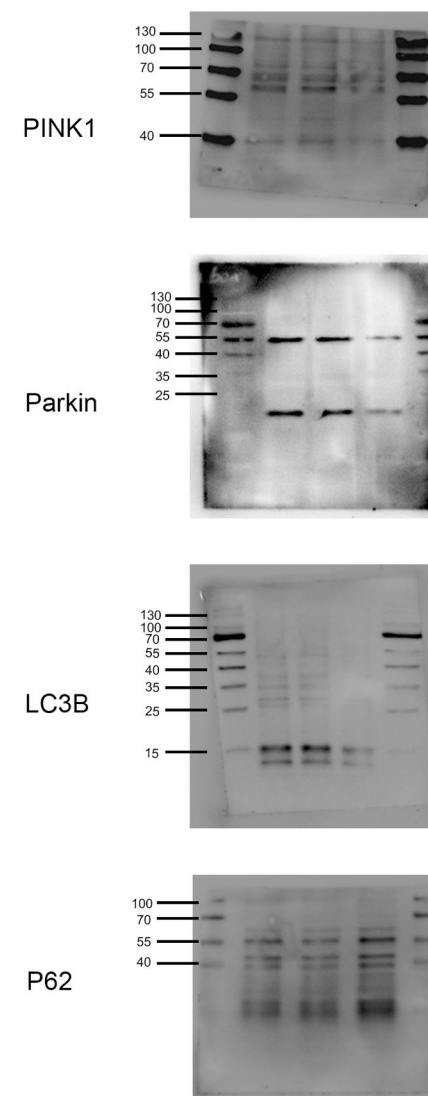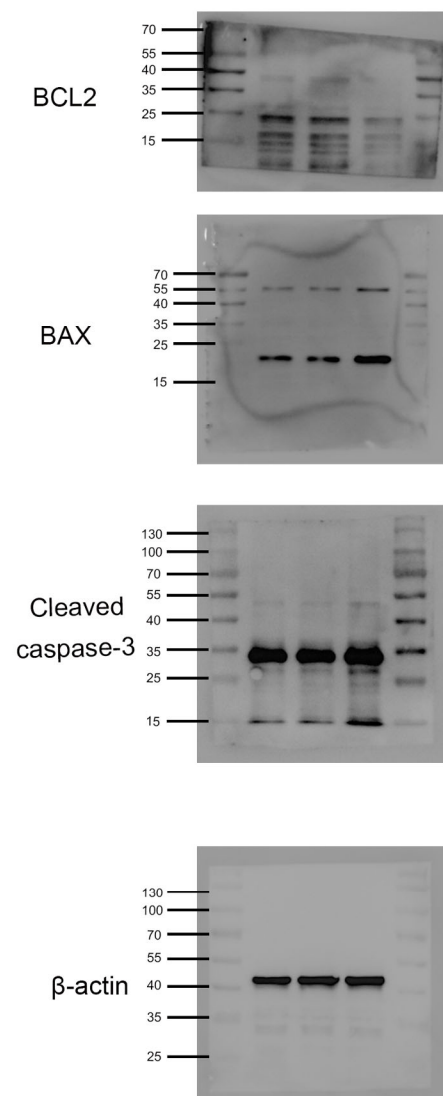

Supplement: Supplementary file 3 — Additional file 2. [file 12964_2023_1399_MOESM2_ESM.pdf]
