# Supplementary material for: ALCAT1-mediated abnormal cardiolipin remodelling promotes mitochondrial injury in podocytes in diabetic kidney disease
Source: Cell Commun Signal. 2024 Jan 10;22:26. doi: 10.1186/s12964-023-01399-4 (PMC10777643; doi:10.1186/s12964-023-01399-4)

Dear editors/reviewers,

The uncropped blots were shown side by side in the same figures.Thank you for your work.

Fig2C

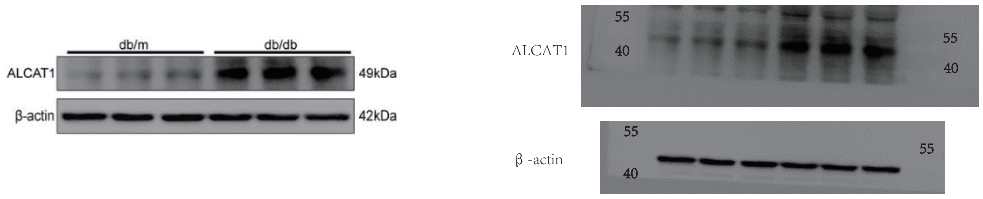

Fig3E

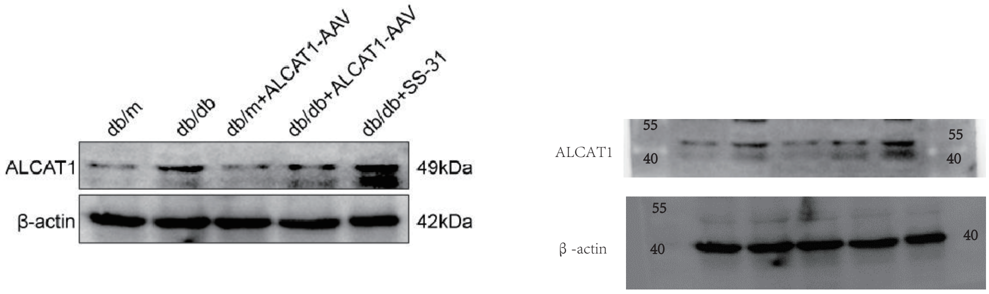

Fig4D

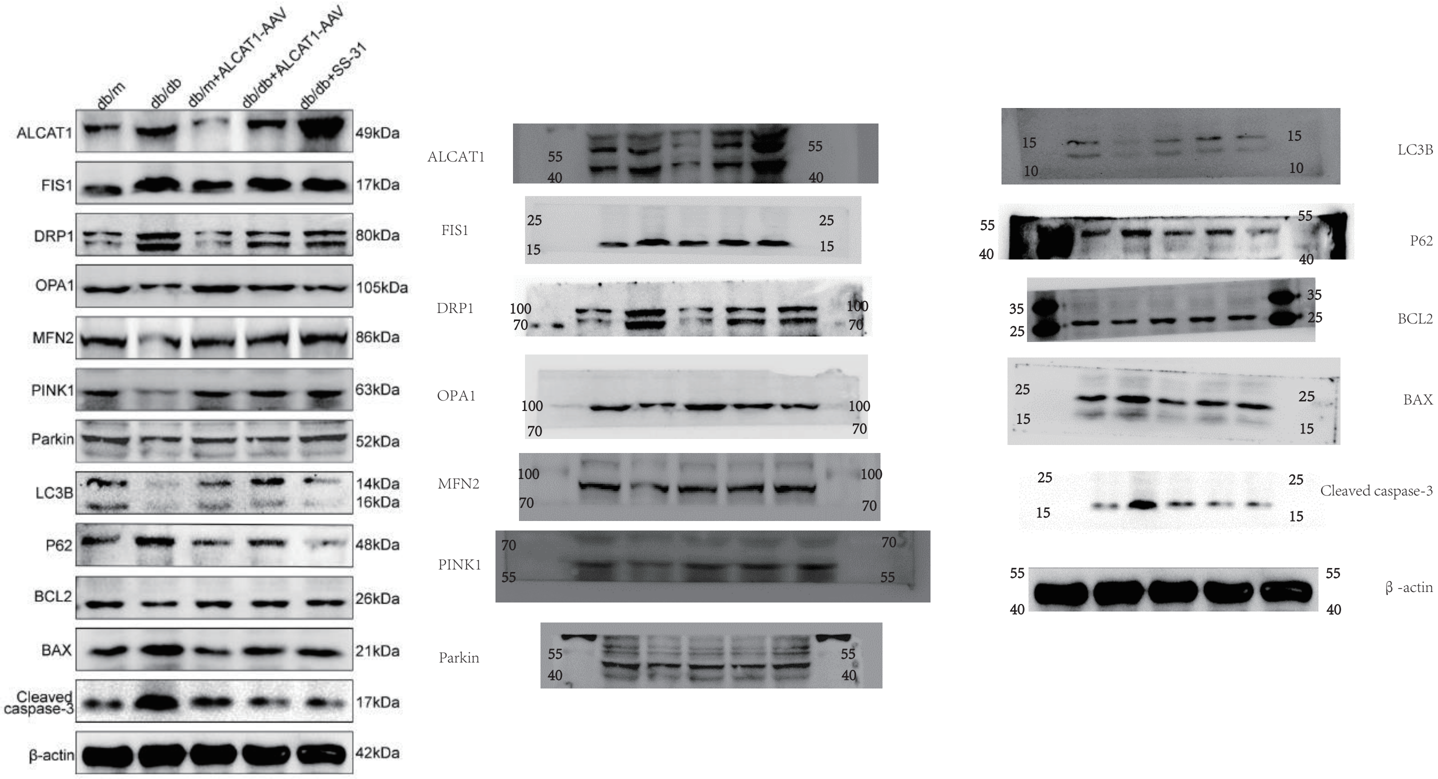

Fig5A

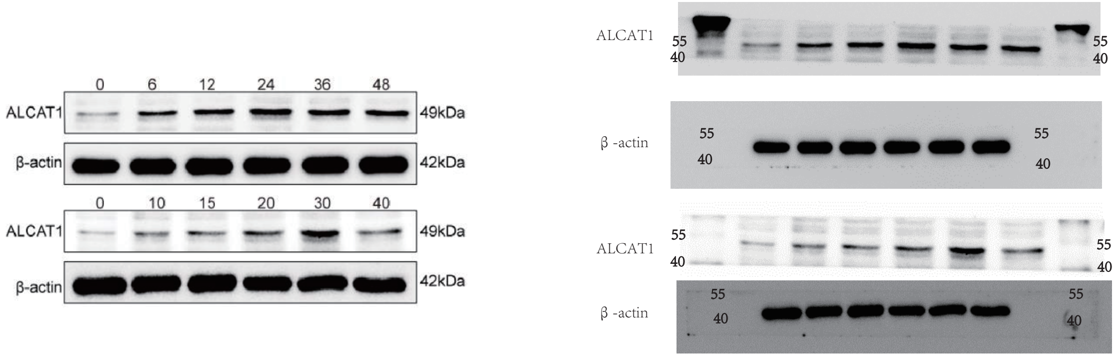
0 6 12 24 36 48
ALCAT1 49kDa
β-actin 42kDa
0 10 15 20 30 40
ALCAT1 49kDa
β-actin 42kDa
ALCAT1
β -actin
ALCAT1
β -actin
55
40

Fig5J

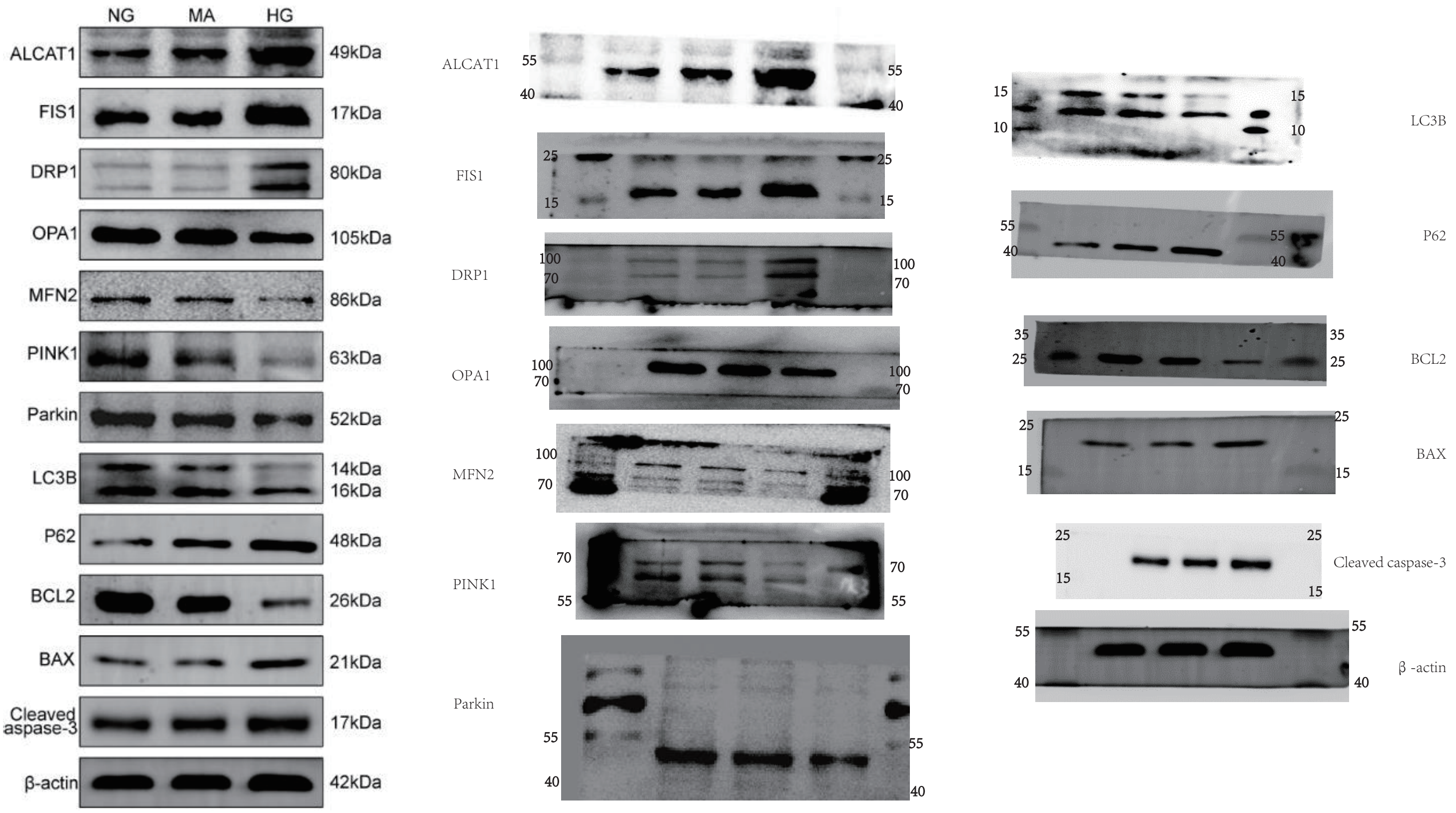
NG MA HG
ALCAT1 49kDa
FIS1 17kDa
DRP1 80kDa
OPA1 105kDa
MFN2 86kDa
PINK1 63kDa
Parkin 52kDa
LC3B 14kDa 16kDa
P62 48kDa
BCL2 26kDa
BAX 21kDa
Cleaved aspase-3 17kDa
β-actin 42kDa
ALCAT1
FIS1
DRP1
OPA1
MFN2
PINK1
Parkin
LC3B
P62
BCL2
BAX
Cleaved caspase-3
β -actin

Fig6A

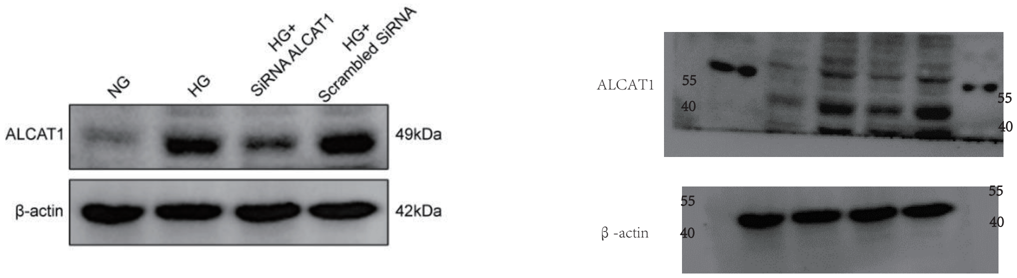

NG
HG
HG+ SiRNA ALCAT1
HG+ Scrambled SiRNA
ALCAT1
49kDa
β-actin
42kDa
ALCAT1
55
40
55
40
β -actin
55
40
55
40

Fig6J

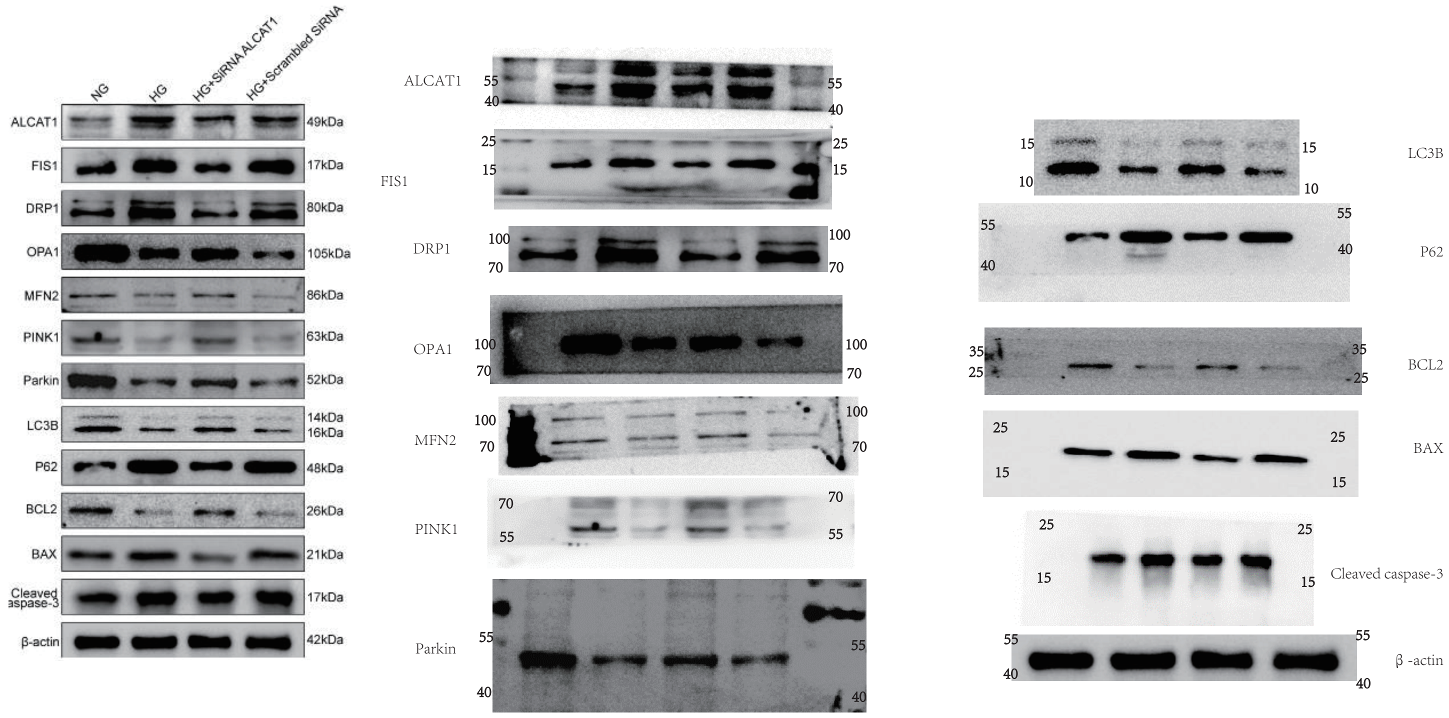

NG
HG
HG+SiRNA ALCAT1
HG+Scrambled SiRNA
ALCAT1
49kDa
FIS1
17kDa
DRP1
80kDa
OPA1
105kDa
MFN2
86kDa
PINK1
63kDa
Parkin
52kDa
LC3B
14kDa
16kDa
P62
48kDa
BCL2
26kDa
BAX
21kDa
Cleaved caspase-3
17kDa
β-actin
42kDa
ALCAT1
FIS1
DRP1
OPA1
MFN2
PINK1
Parkin
LC3B
P62
BCL2
BAX
Cleaved caspase-3
β -actin

Fig7A

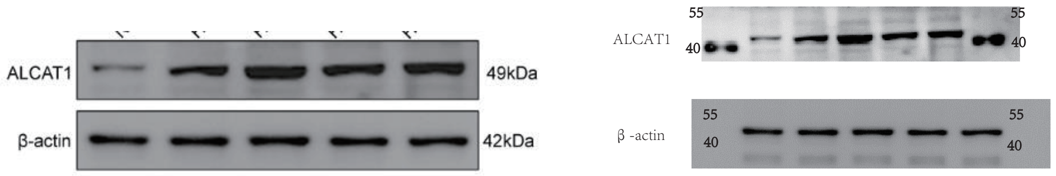

ALCAT1
49kDa
β-actin
42kDa
55
40

Fig7J

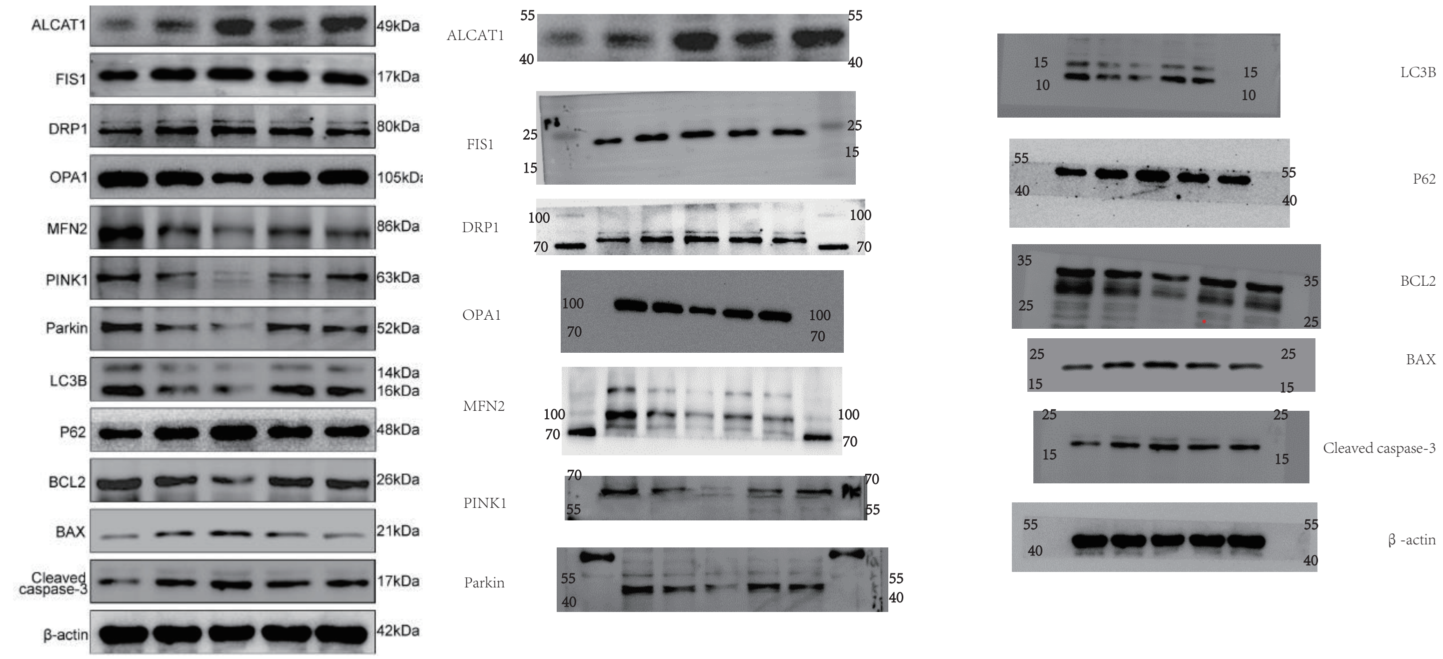

ALCAT1
49kDa
FIS1
17kDa
DRP1
80kDa
OPA1
105kDa
MFN2
86kDa
PINK1
63kDa
Parkin
52kDa
LC3B
14kDa
16kDa
P62
48kDa
BCL2
26kDa
BAX
21kDa
Cleaved caspase-3
17kDa
β-actin
42kDa

Fig8A

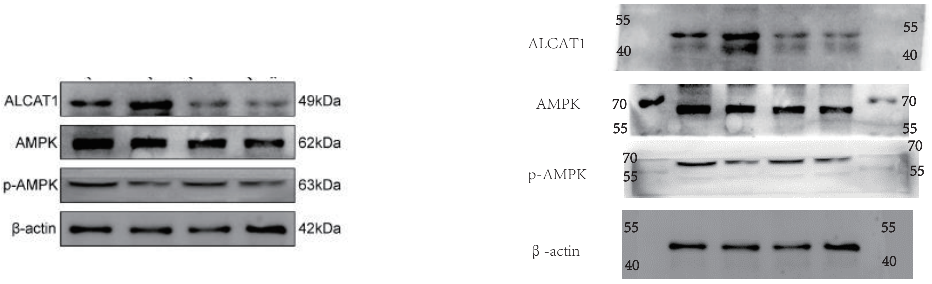

Fig8B

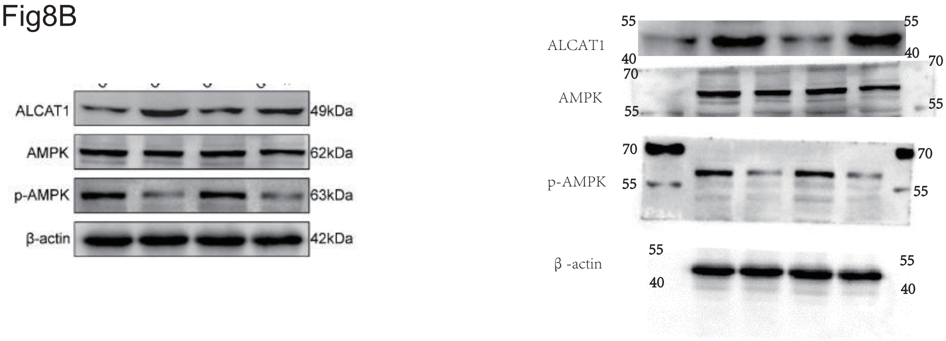

Fig8C

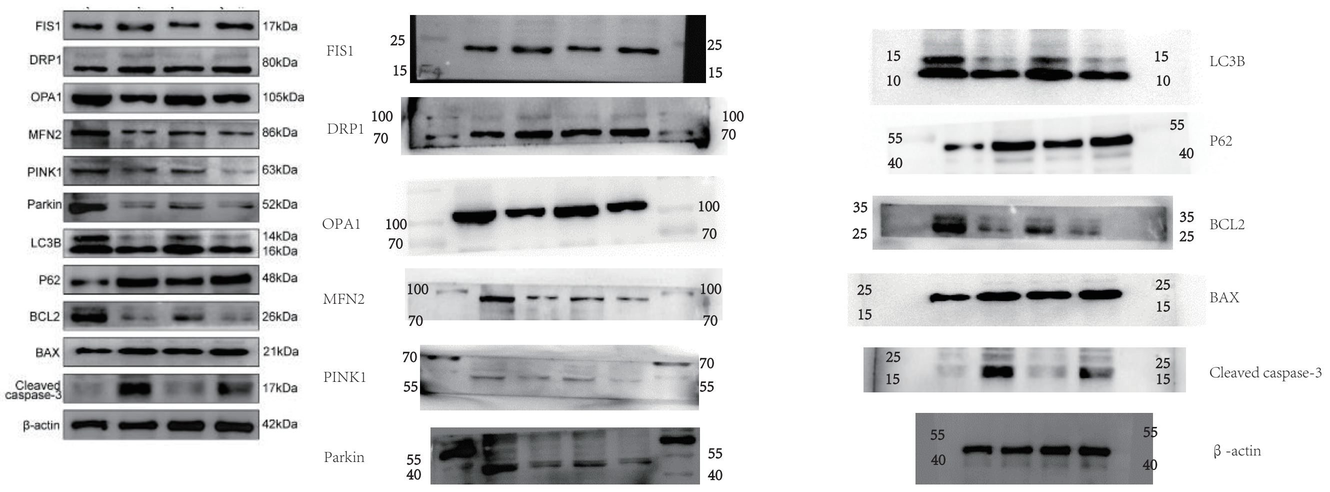

Fig8D

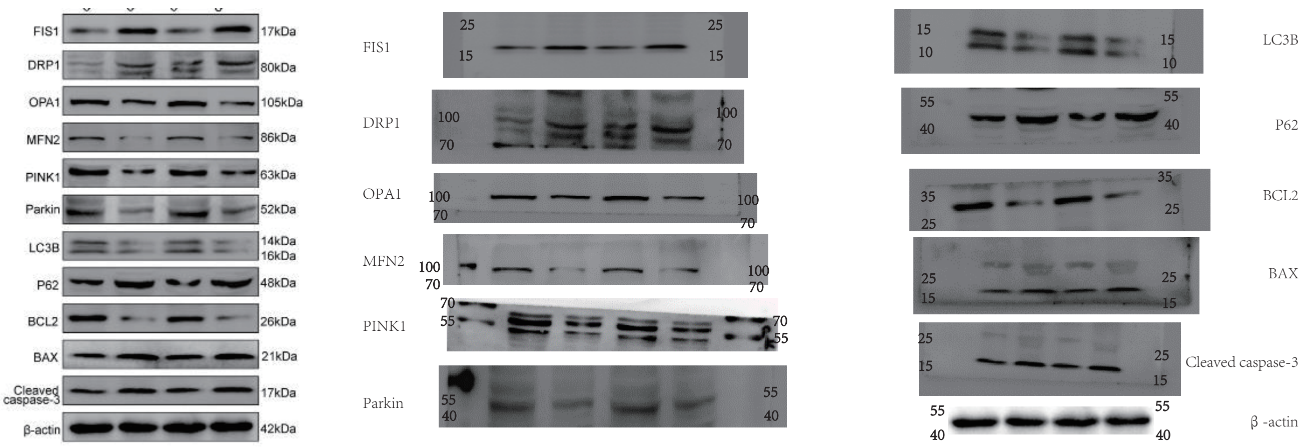
FIS1 17kDa
DRP1 80kDa
OPA1 105kDa
MFN2 86kDa
PINK1 63kDa
Parkin 52kDa
LC3B 14kDa 16kDa
P62 48kDa
BCL2 26kDa
BAX 21kDa
Cleaved caspase-3 17kDa
β-actin 42kDa
FIS1 25 15
DRP1 100 70
OPA1 100 70
MFN2 100 70
PINK1 70 55
Parkin 55 40
LC3B 15 10
P62 55 40
BCL2 35 25
BAX 25 15
Cleaved caspase-3 25 15
β -actin 55 40

Supplementary Fig.2C

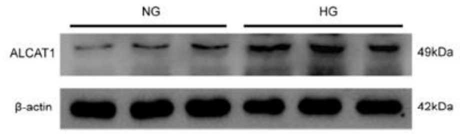

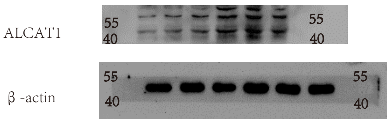

Supplementary Fig.3

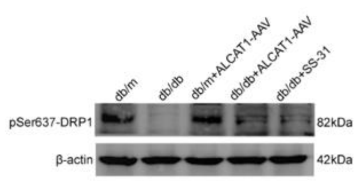

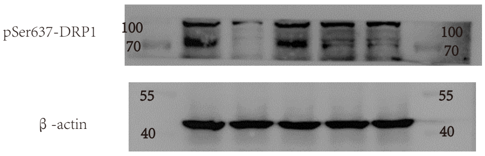

Supplementary Fig.4

A

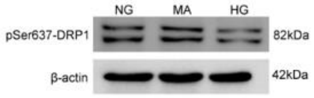

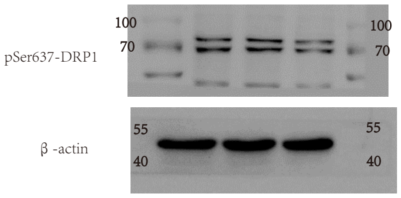

B

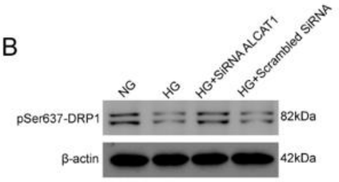

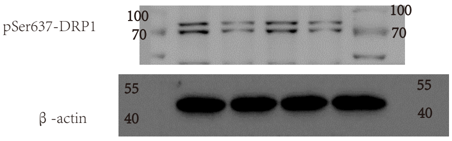

C

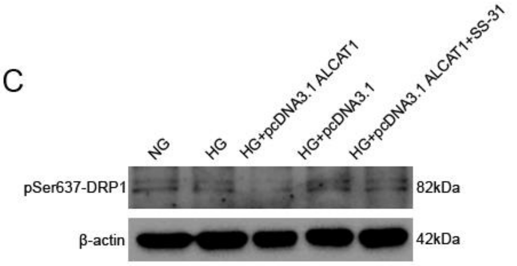

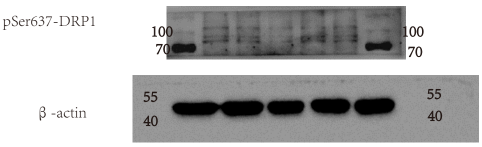

Supplementary Fig.5

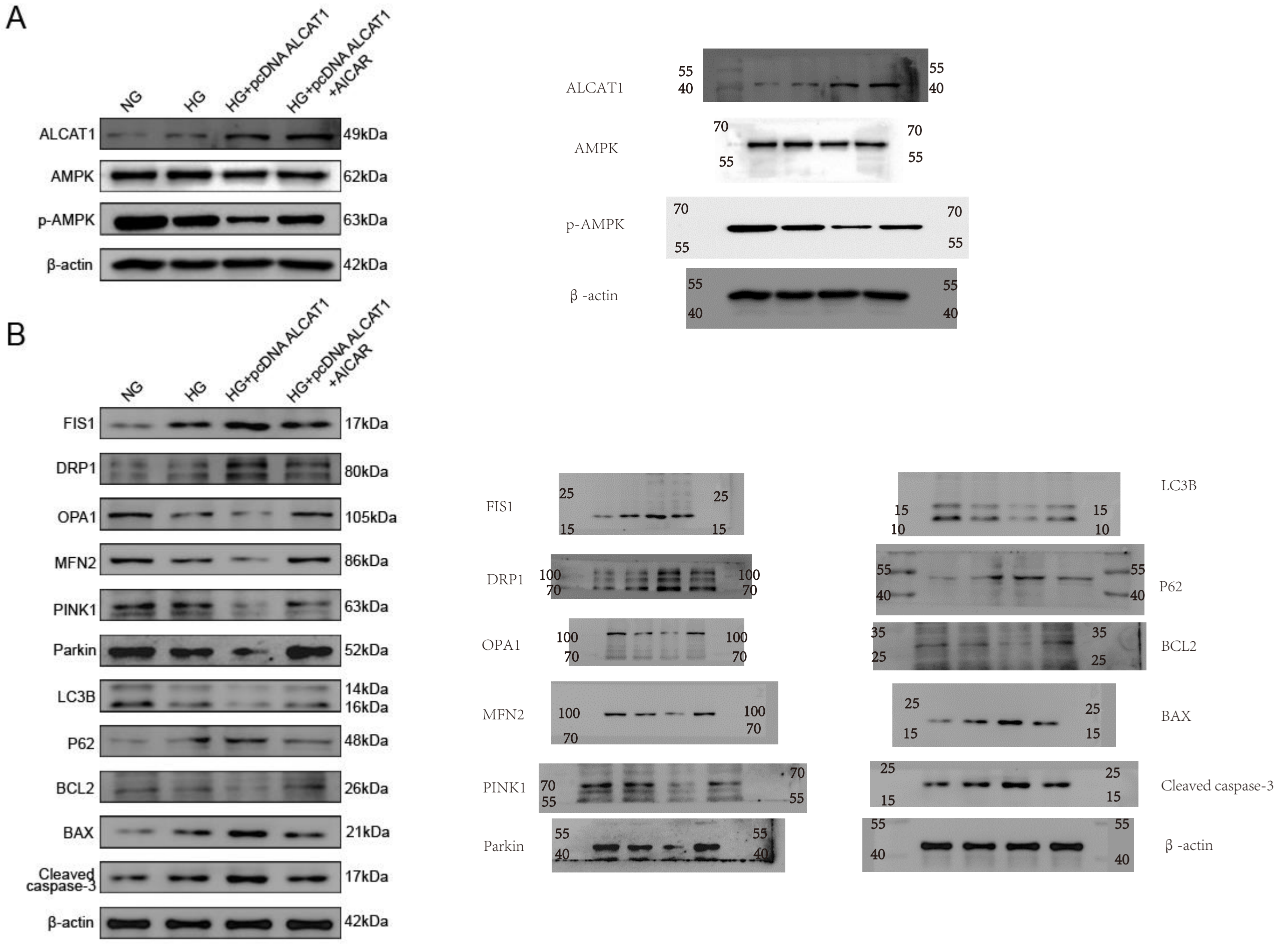

Supplement: Supplementary file 4 — Additional file 3. [file 12964_2023_1399_MOESM3_ESM.pdf]
